# Supplementary material for: Site-specific characterization of endogenous SUMOylation across species and organs
Source: Nat Commun. 2018 Jun 25;9:2456. doi: 10.1038/s41467-018-04957-4 (PMC6018634; doi:10.1038/s41467-018-04957-4)
Supplement: Supplementary file 1 — Supplementary Information [file 41467_2018_4957_MOESM1_ESM.pdf]

SUPPLEMENTARY INFORMATION

Site-specific characterization of endogenous SUMOylation across species and organs

Hendriks et al.

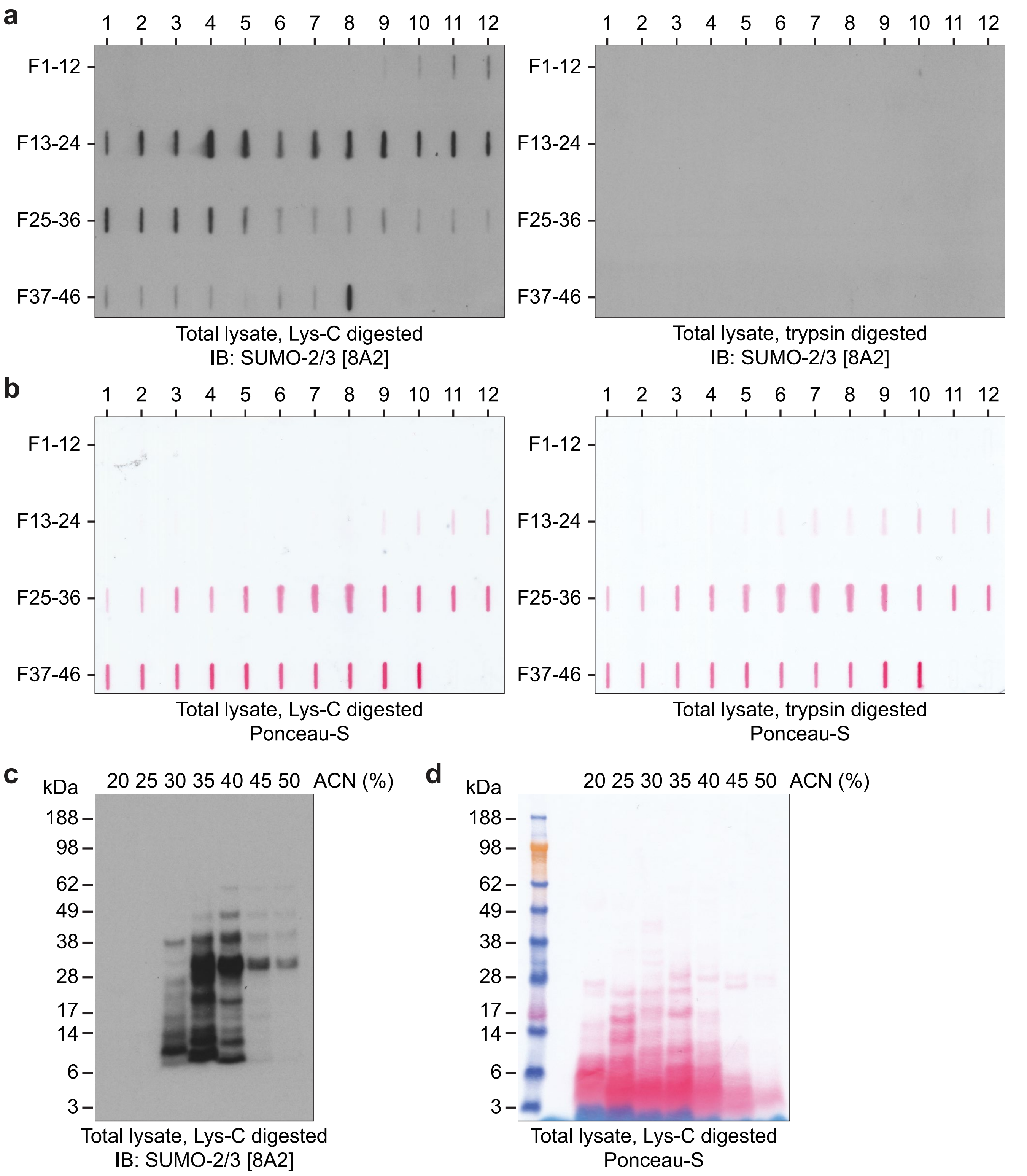

Supplementary Figure 1

Supplementary Figure 1. Verification of the 8A2 antibody and immunoprecipitation. (A) Dot blot analysis of HeLa total lysate, digested either with endopeptidase Lys-C (left) or trypsin (right), and fractionated into 46 fractions using high-pH reversed-phase chromatography. Immunoblot was performed using the 8A2 antibody. (B) Ponceau-S analysis of the membranes displayed in **A,** to visualize total peptide content. (C) Immunoblot analysis of Lys-C digested HEK lysate, as eluted off C8 SepPak cartridges using increasing percentages of acetonitrile (ACN). Immunoblot was performed using the 8A2 antibody. (D) Ponceau-S analysis of the membrane displayed in **C**, to visualize total peptide content.

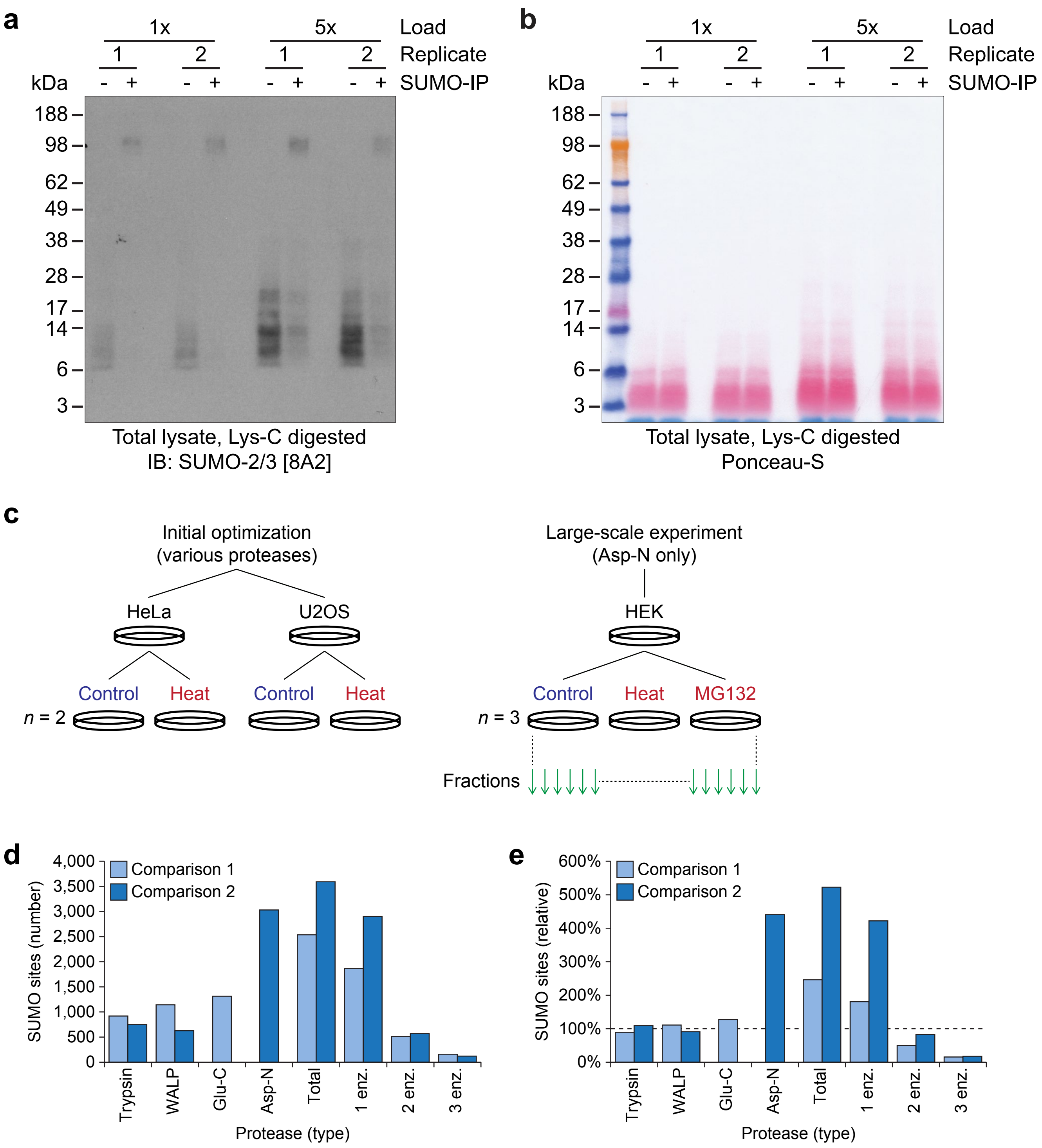

Supplementary Figure 2

Supplementary Figure 2. Optimization of the SUMO-IP method. (A) Immunoblot analysis of SUMO-immunoprecipitation (IP) performed using the 8A2 antibody, from Lys-C digested HeLa lysate, after desalting the peptides. Immunoblot was performed using the 8A2 antibody. (B) Ponceau-S analysis of the membrane displayed in **A**, to visualize total peptide content. (C) Overview of the initial experimental design used to evaluate the performance of different second-stage proteolytic digestion enzymes, alongside the final experimental setup used to benchmark the method at larger scale. (D) Overview of the number of SUMO2/3 sites identified in the 1st and 2nd rounds of initial optimization, where SUMOylated peptides purified after Lys-C digest were split into three pools and either digested with trypsin, WALP, and Glu-C (1st round), or trypsin, WALP, and Asp-N (2nd round). “Enz.”, SUMO2/3 sites identified by the indicated number of enzymes. (E) As **D**, but with the number of sites normalized to trypsin and WALP, as these two enzymes were used in both optimization rounds.

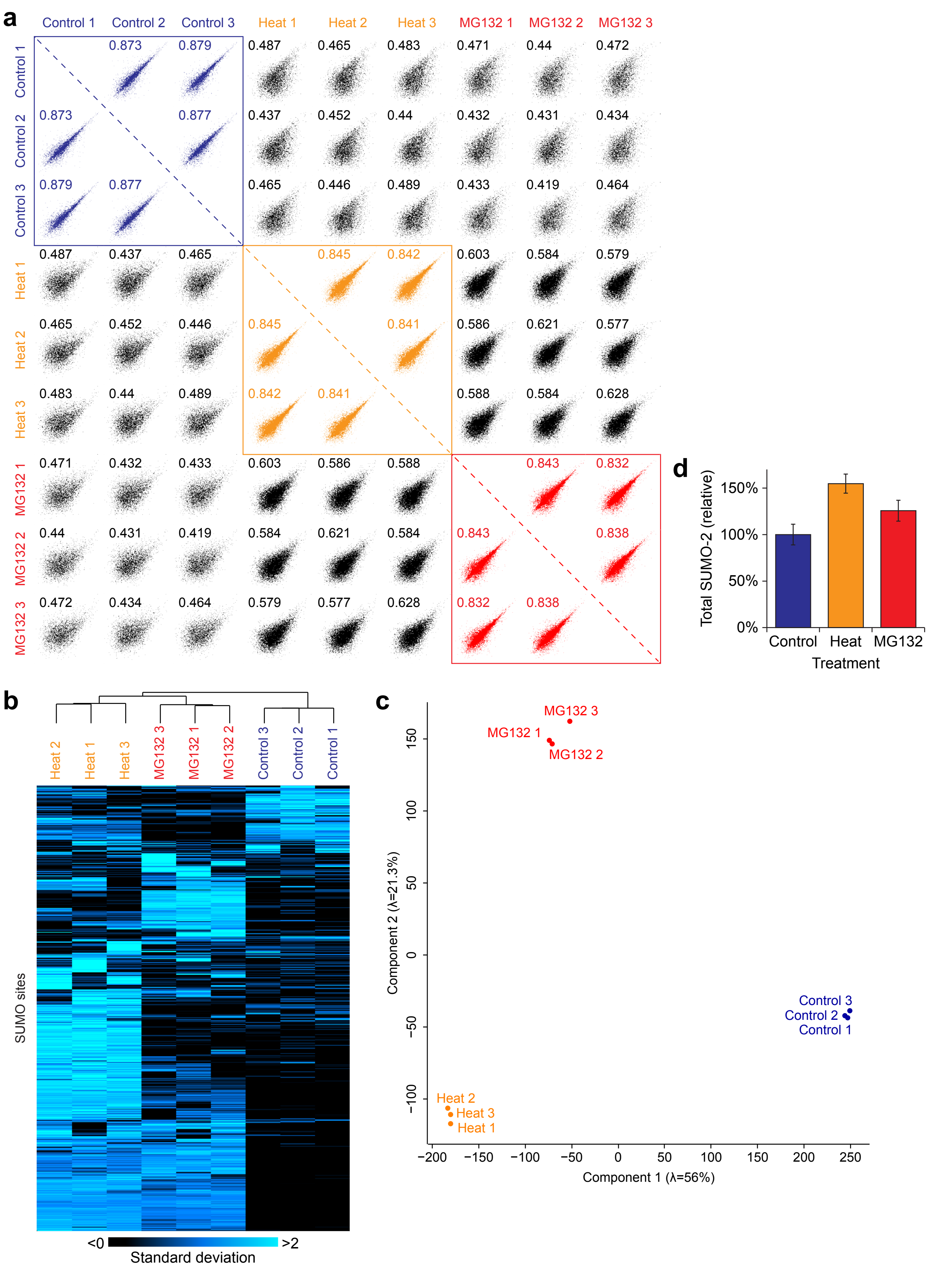

Supplementary Figure 3

Supplementary Figure 3. Method reproducibility assessment in HEK cells. (A) Scatter plot analysis of log_2 transformed intensity values corresponding to SUMO2/3 sites identified in all HEK experiments, demonstrating reproducibility between same-condition replicates. Values indicate Pearson correlation. (B) Hierarchical clustering analysis of z-scored log_2 transformed intensity values corresponding to all identified SUMO2/3 sites, highlighting clusters of consistently identified sites across same-condition replicates. Blue coloring is indicative of relative presence; black denotes 0 or less SD, blue 1 SD, cyan 2 or more SD. *n*=3 cell culture replicates. (C) Principle component analysis (PCA) of all HEK experiments at the SUMO2/3 sites level, visualizing the maximum degree of variability observed between all experiments. Eigenvalues are displayed on the axes. (D) Quantification of internal SUMO-2/3 peptides, AYCERQGLSMRQIRFRF and AYCERQGLSMRQIRFRFDGQPINET, corresponding to total SUMO-2/3 purified from HEK cells after Lys-C digest. Error bars represent SEM, *n*=6 peptide comparisons derived from two peptides and three cell culture replicates.

Human SUMO motifs (Motif-X, $p < 10^{-6}$)

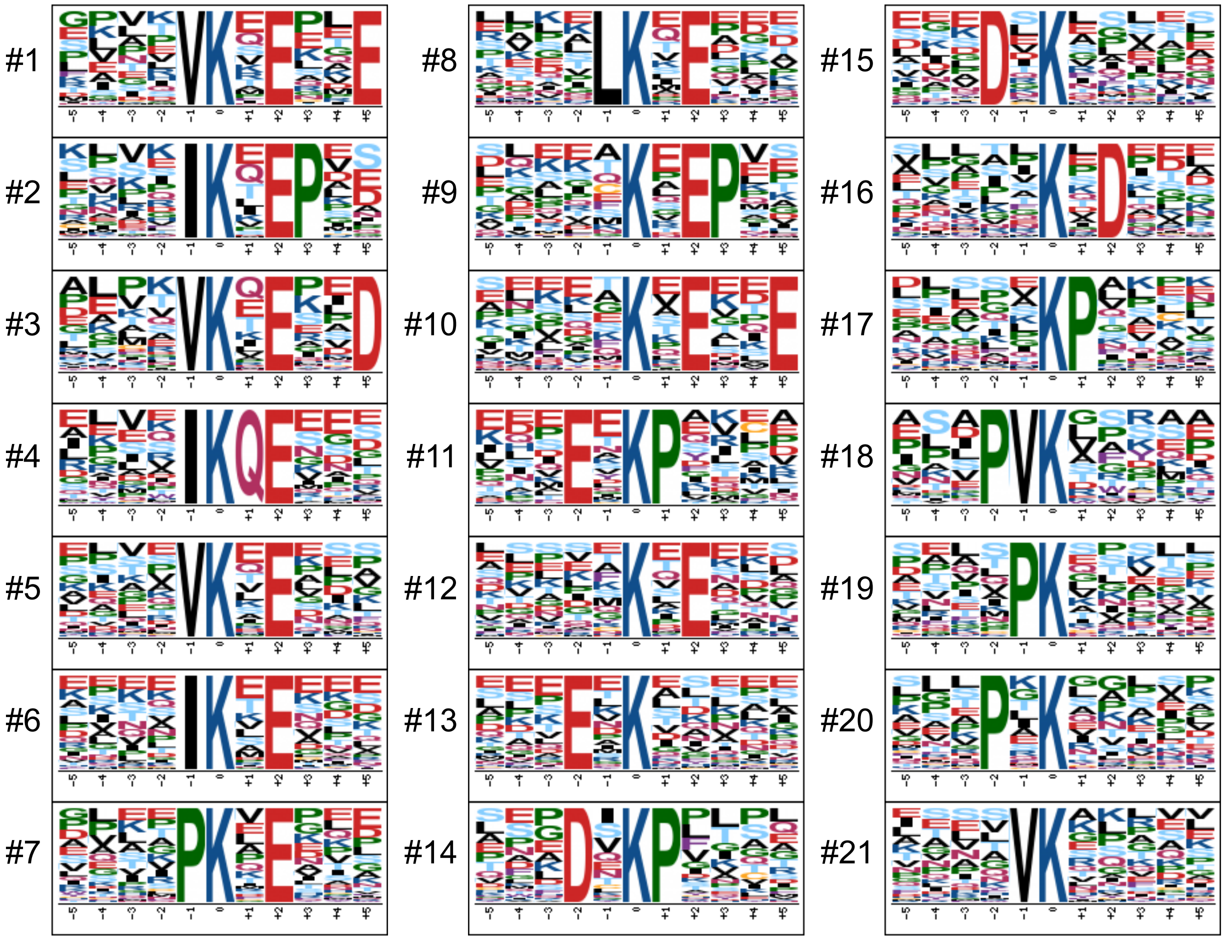

Supplementary Figure 4

Supplementary Figure 4. Motif-X analysis of SUMO consensus motifs in cultured human cells. Motif-X analysis of 11 amino acid sequence windows centered on SUMO-modified lysines corresponding to all SUMO sites detected in human cells under standard growth conditions. Default Motif-X settings were used, with significance filtering of $p < 10^{-6}$. All identified motifs and their statistics, as well as an analysis on all SUMO sites detected in human cells including those identified in response to stress, are available in Supplementary Data 1.

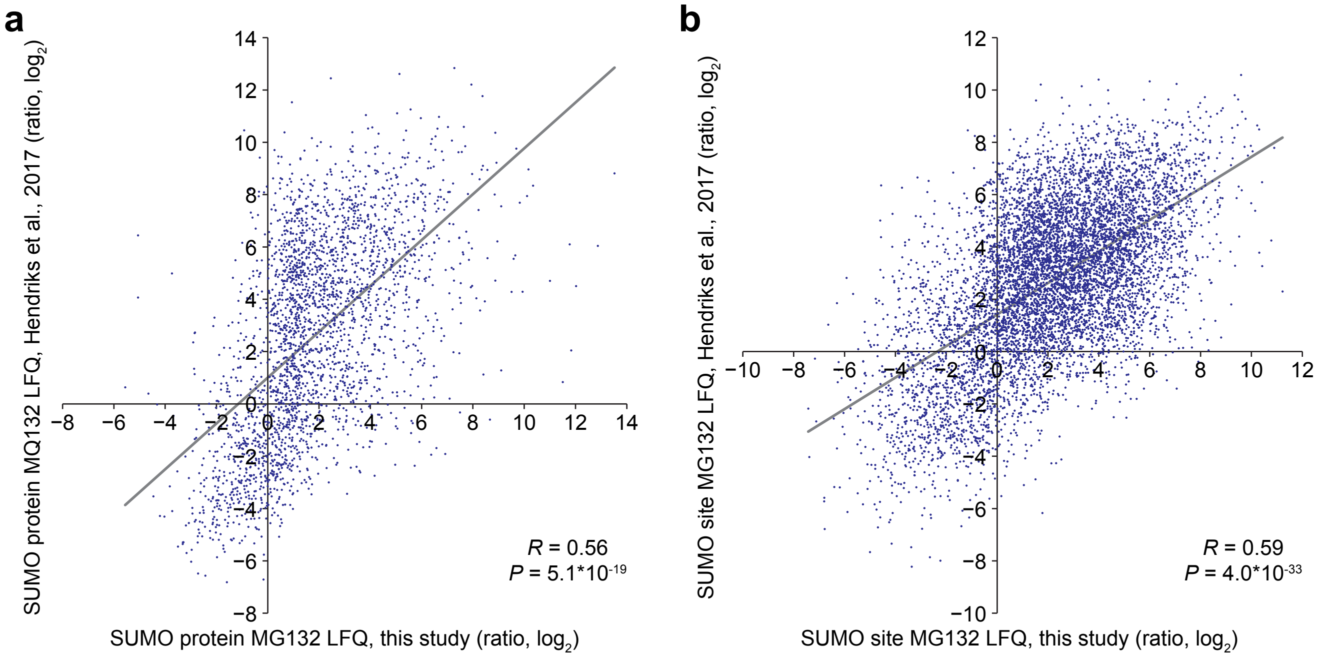

Supplementary Figure 5

Supplementary Figure 5. Comparison of the SUMO stress response between endogenous and exogenous studies. (A) Scatter plot analysis of SUMO2/3 target proteins that were LFQ quantified in response to MG132 treatment, in this study (HEK cells) compared to a study using exogenous K0-SUMO (HeLa cells)[1]. Every dot represents a protein quantified in both studies. The line represents the best-fit linear correlation, with the Pearson correlation (*R*) and *p*-value indicated. The *p*-value was determined by two-tailed Student's *t*-testing, *n*=2,674 quantified protein ratio pairs. (B) As **A**, but for SUMO2/3 sites LFQ quantified in response to MG132 treatment in both studies. *n*=7,534 quantified site ratio pairs.

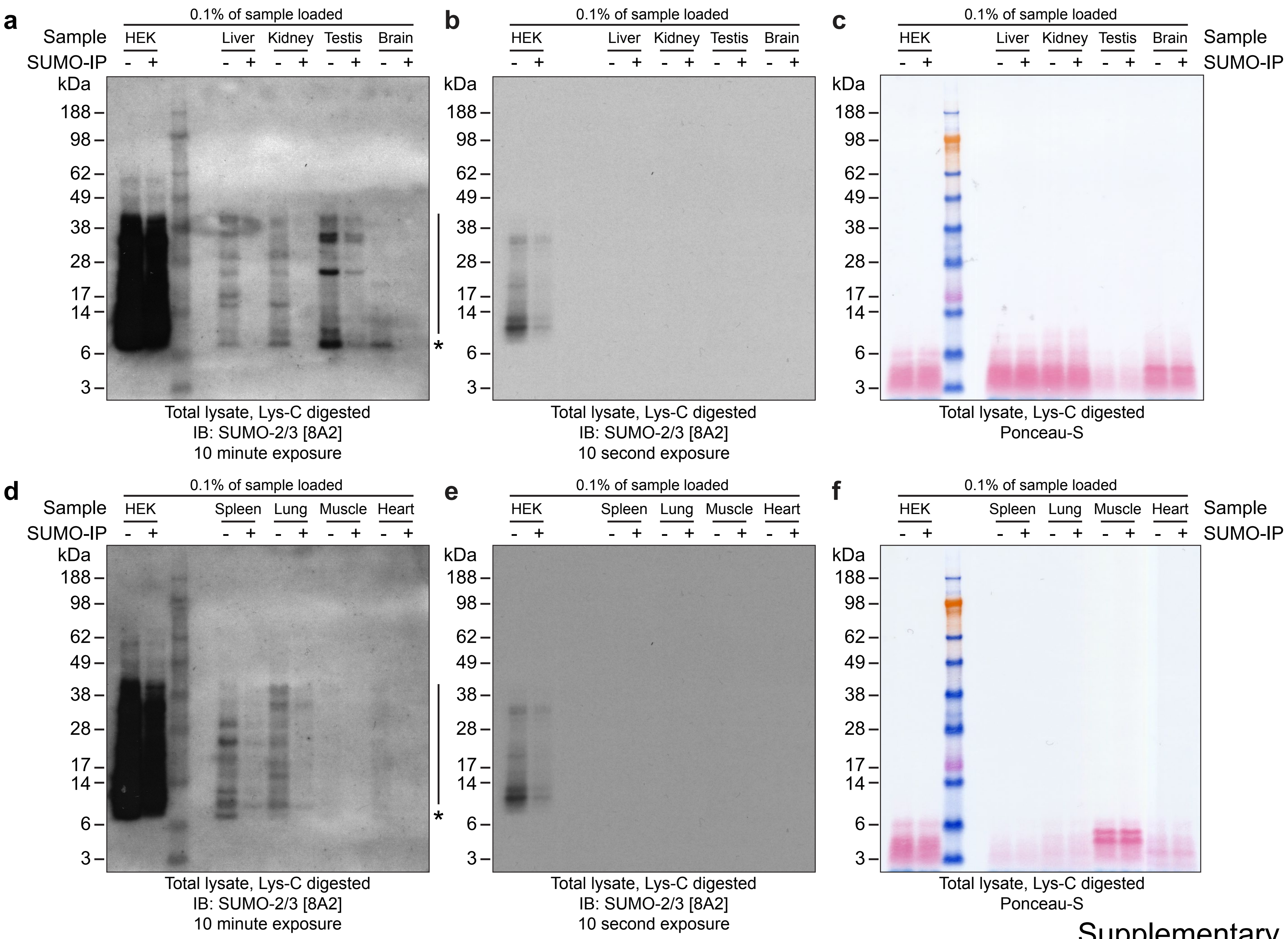

Supplementary Figure 6

Supplementary Figure 6. Verification of SUMO-IP efficiency from mouse organs. (A) Immunoblot analysis of SUMO-immunoprecipitation performed using the 8A2 antibody, from Lys-C digested mouse organs (liver, kidney, testis, and brain), after desalting the peptides. An amount of Lys-C digest corresponding to 0.1% of an entire organ was loaded (excepting liver for which half the organ was used), or 0.1% of the Lys-C digest corresponding to the protein content of one HEK cell culture replicate (50 mg). Immunoblot probing was performed using the 8A2 antibody; long exposure. The asterisk indicates the fragment resulting from Lys-C digestion of free SUMO, whereas the black line indicates the same mass remnant linked to various target peptides, resulting from Lys-C digestion of conjugated SUMO. (B) As **A**, but a short exposure. (C) Ponceau-S analysis of the membrane displayed in **A** and **B**, to visualize total peptide content. (D) As **A**, but with analysis performed on spleen, lung, muscle, and heart. (E) As **D**, but a short exposure. (F) Ponceau-S analysis of the membrane displayed in **D** and **E**, to visualize total peptide content.

Mouse SUMO motifs (Motif-X, $p < 10^{-6}$)

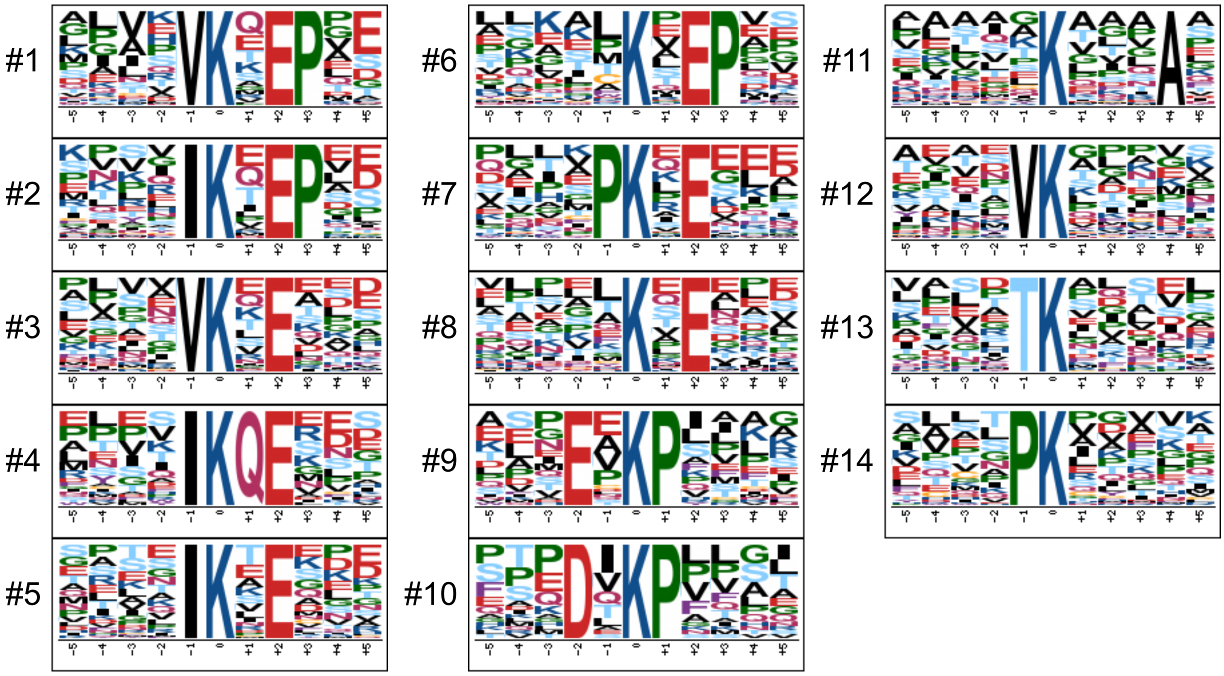

Supplementary Figure 7

Supplementary Figure 7. Motif-X analysis of SUMO consensus motifs in mouse organs. Motif-X analysis of 11 amino acid sequence windows centered on SUMO-modified lysines corresponding to all SUMO sites detected across eight mouse organs. Default Motif-X settings were used, with significance filtering of $p < 10^{-6}$. All identified motifs and their statistics are available in Supplementary Data 5.

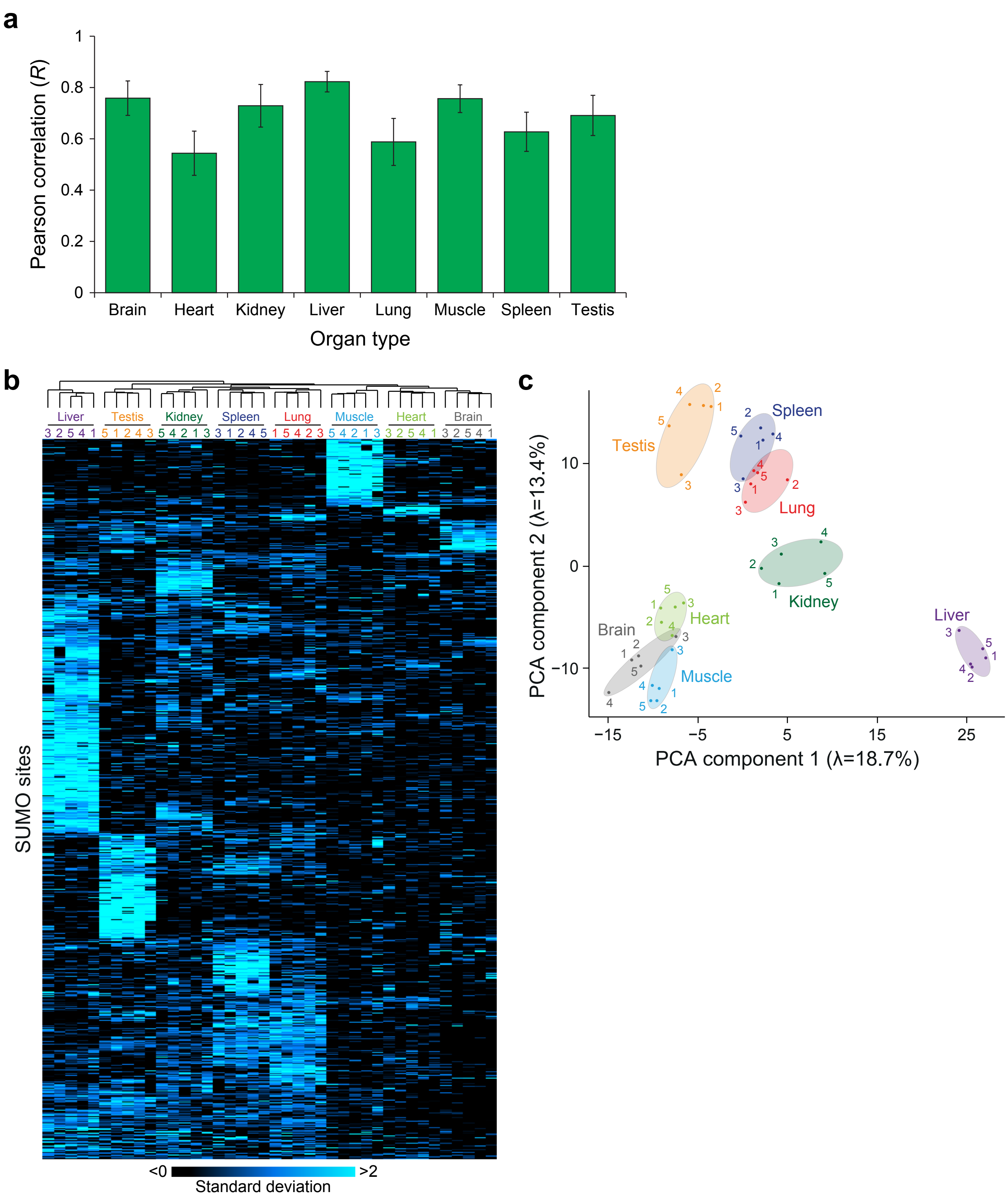

Supplementary Figure 8

Supplementary Figure 8. Method reproducibility assessment in mouse organs. (A) Average Pearson correlation observed between same-organ replicates, derived from scatter plot analysis of log_2 transformed intensity values corresponding to SUMO2/3 sites. Error bars represent SD, *n*=5 animals. (B) Hierarchical clustering analysis of z-scored label-free quantified (LFQ) expression values corresponding to mouse SUMO2/3 sites detected across replicates and organs. Blue coloring indicates relative presence in a sample as compared to others. *n*=5 animals. (C) Principle component analysis (PCA) of all mouse experiments, at the SUMO2/3 sites level. Eigenvalues are displayed on the axes.

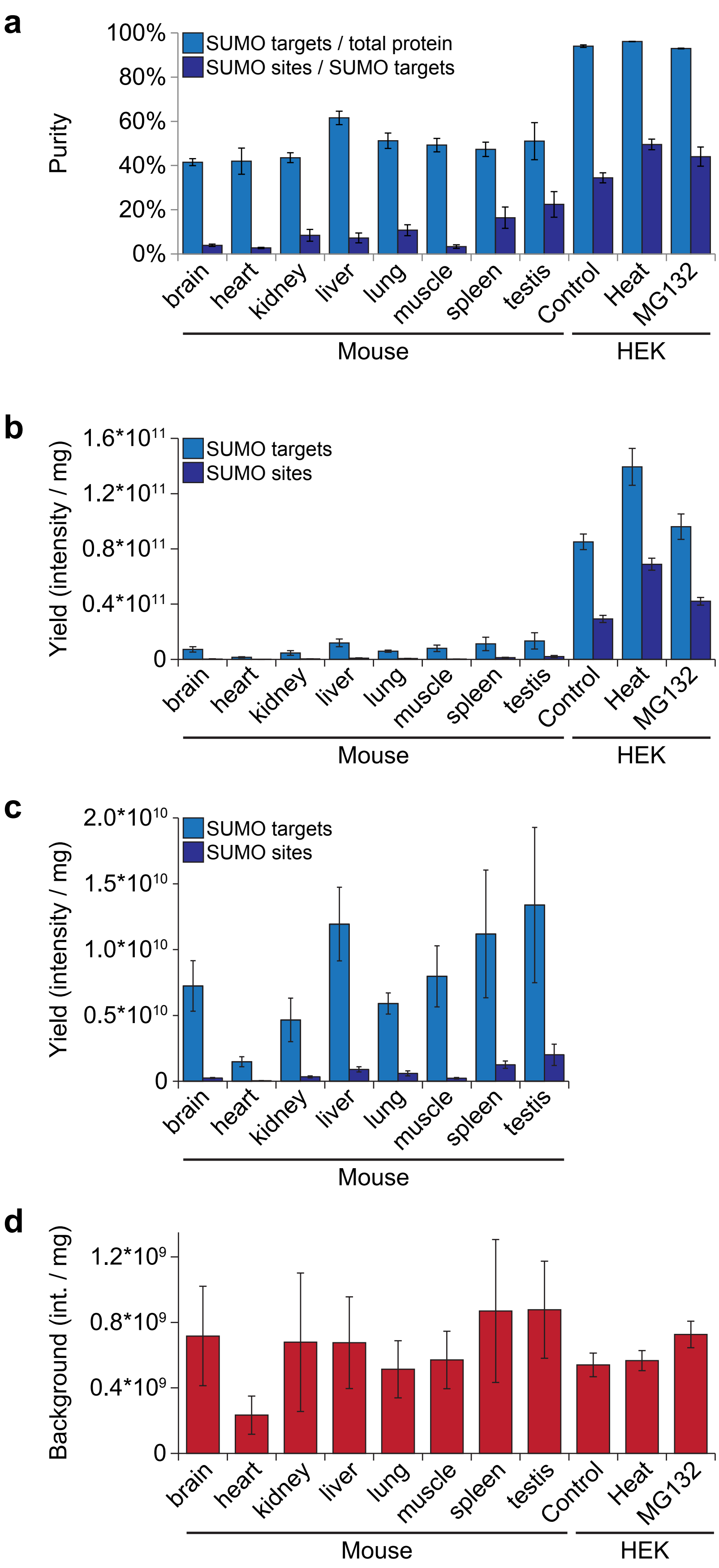

Supplementary Figure 9

Supplementary Figure 9. Determination of purity and yield. (A) Schematic overview of the purity of samples derived from mouse organs and human cell lines. Overall purity is represented as the fraction of SUMO2/3 target proteins obtained after SUMO-IP on Lys-C digested material. Site-level purity is represented as the fraction of modified peptides versus unmodified peptides identified in SUMO2/3 target proteins. Error bars represent SD, *n*=5 animals for the mouse data, and *n*=3 cell culture replicates for the human data. (B) Overview of the SUMO2/3 target protein and SUMO2/3 site yield obtained from mouse organs and human cell lines, per milligram of total protein in the starting material. Yield is depicted as the overall signal intensity obtained while measuring samples on the same mass spectrometer, which is a dimensionless variable that can only be relatively compared. Error bars represent SD, *n*=5 animals for the mouse data, and *n*=3 cell culture replicates for the human data. (C) As **B**, but 10× zoomed on the mouse data. (D) Overview of the background intensity signal, i.e. peptides not corresponding to proteins containing at least one SUMO2/3 site, identified in mouse organs and human cell lines, per milligram of total protein in the starting material. Error bars represent SD, *n*=5 animals for the mouse data, and *n*=3 cell culture replicates for the human data.

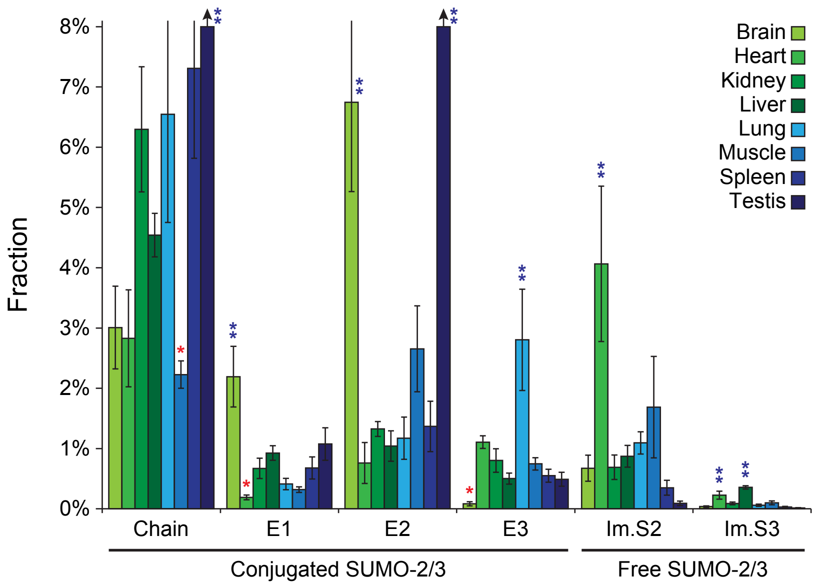

Supplementary Figure 10

Supplementary Figure 10. Insight into the SUMO equilibrium. As **Fig. 7C**, but 10× zoomed on the six low-abundant categories. Error bars represent SEM, n=5 animals. Asterisks indicate significant differences (blue, higher; red, lower) between the indicated organ and the six median organs within the same category, by two-tailed Student's t-test. ** $P<0.001$, * $P<0.05$.

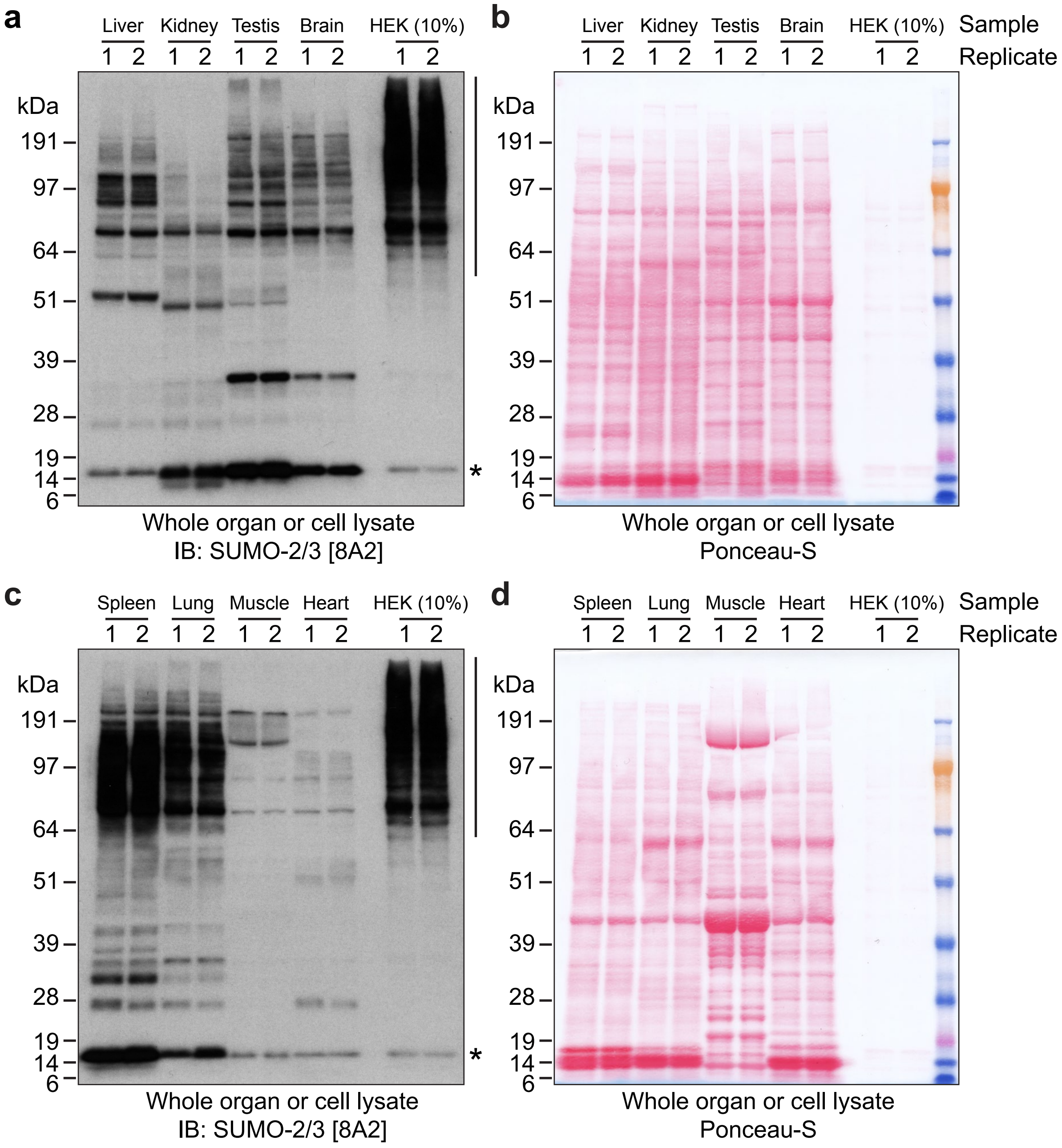

Supplementary Figure 11

Supplementary Figure 11. Validation of the SUMO equilibrium in cultured cells and organs by immunoblotting. (A) Immunoblot analysis of whole mouse organ extracts and total HEK cell lysates, using the 8A2 antibody. Samples were prepared using highly denaturing buffer conditions and potent SUMO protease inhibitors in order to preserve the native SUMO equilibrium[2]. For liver, kidney, testis, and brain, 20 μg of proteins were loaded per lane, whereas only 2 μg of HEK total lysate proteins were loaded per lane. The asterisk indicates free SUMO2/3, whereas the black line indicates SUMO2/3 conjugated to various target proteins. Replicates indicate different animals for organs, and replicate cell cultures for HEK cells. (B) Ponceau-S analysis of the membrane displayed in **A**, to visualize total protein content. (C) As **A**, but with analysis performed on spleen, lung, muscle, and heart. (D) Ponceau-S analysis of the membrane displayed in **C**, to visualize total protein content.

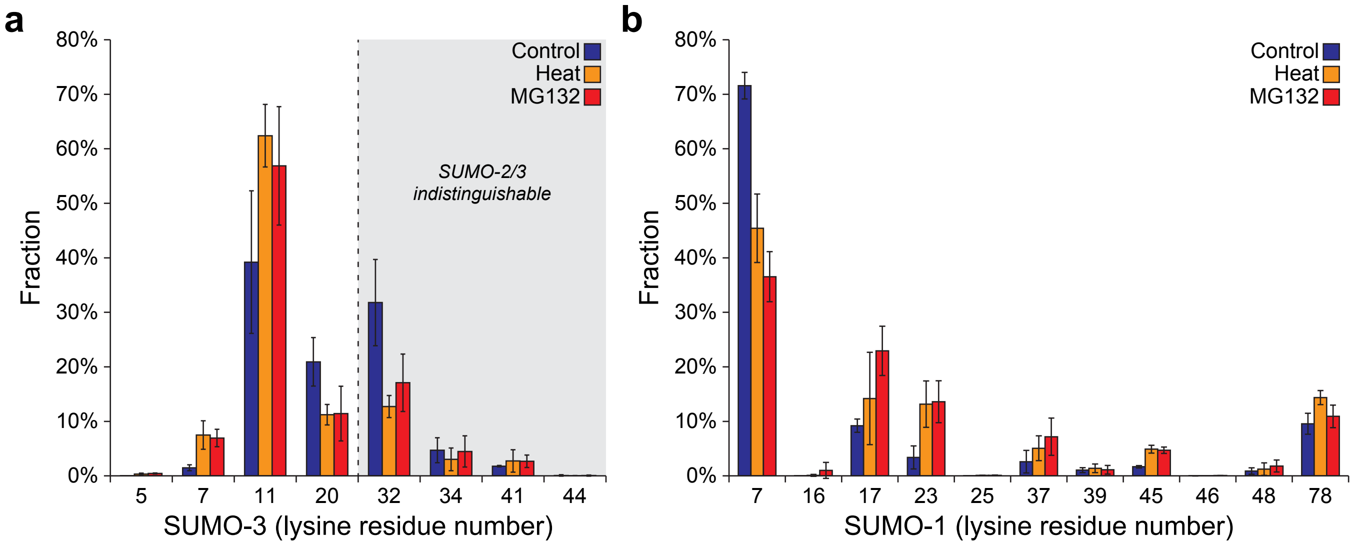

Supplementary Figure 12

Supplementary Figure 12. Insight into SUMO chain architecture. (A) As **Fig. 7D**, but quantifying the modification of endogenous SUMO-3 by endogenous SUMO-2/3. Error bars represent SD, *n*=3 cell culture replicates. (B) As **Fig. 7D**, but quantifying the modification of endogenous SUMO-1 by endogenous SUMO-2/3. Error bars represent SD, *n*=3 cell culture replicates.

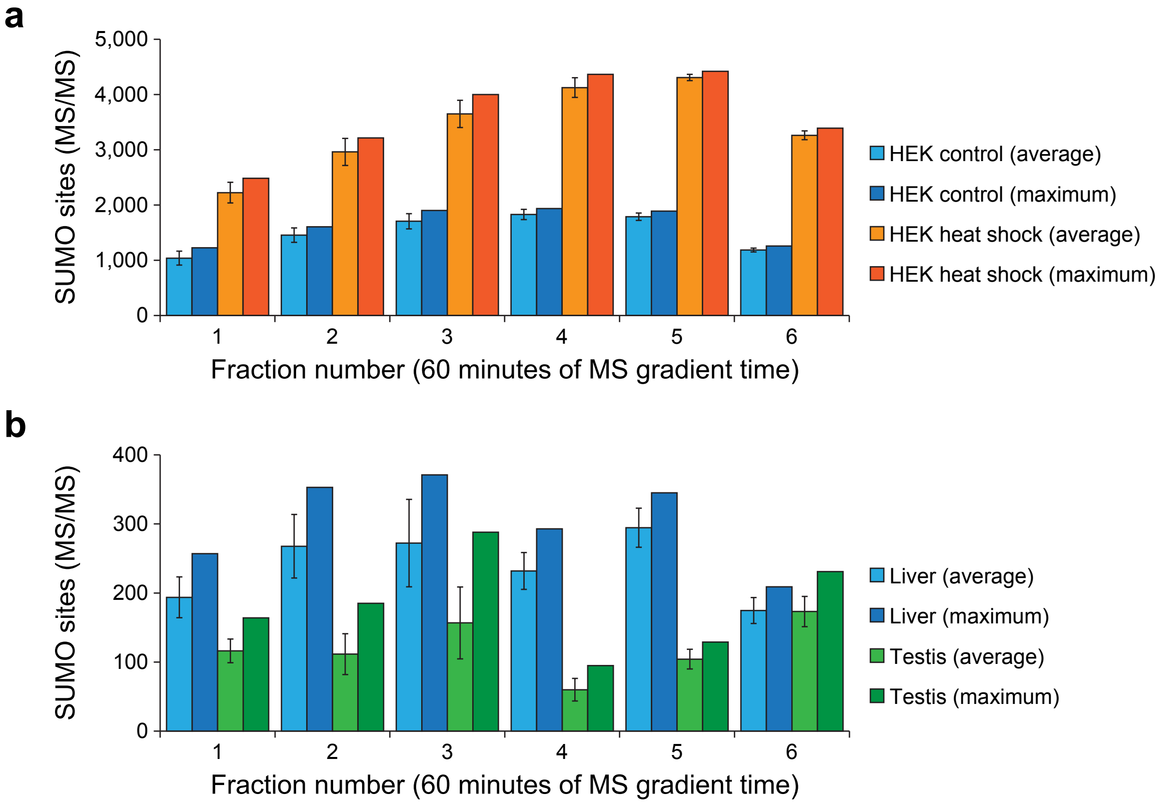

Supplementary Figure 13

Supplementary Figure 13. Evaluation of per-fraction and per-replicate performance of our endogenous SUMO-IP proteomics strategy. (A) Overview of the number of SUMO-2/3 sites identified directly by MS/MS, per single replicate and per single fraction, in HEK cells under standard growth conditions and in response to heat shock. Each fraction corresponds to ~8 mg of starting total protein material, or ~30 million cells. Fractions were measured using 60 minutes of mass spectrometric analysis time (using a Q-Exactive HF instrument), and the numbers of SUMO2/3 sites identified can be achieved in single analyses within this timeframe. Average number of SUMO2/3 sites and the best-replicate number of SUMO2/3 sites are shown. Error bars represent SEM, *n*=3 cell culture replicates. (B) As **A**, but for the mouse liver and testis. Each fraction corresponds to ~1/12th of a mouse liver, or ~1/6th of a pair of mouse testes. Error bars represent SEM, *n*=5 animals.

SUPPLEMENTARY NOTE 1

Optimization of the SUMO-IP

Whereas other SUMO enrichment methods rely on denaturing buffer conditions, or seek to dilute the concentration of denaturants prior to enrichment, we chose to entirely eliminate denaturing agents for the purification phase. Following Lys-C digestion, peptides were desalted on C8 resin to eliminate guanidine from the samples and to efficiently capture larger peptides, as the SUMO mass remnant generated upon Lys-C digestion is 5.6 kDa. Moreover, we investigated at which percentage of solvent SUMOylated peptides elute from the resin (Supplementary Fig. 1C-D), and adapted the protocol to include a highly selective elution range. Importantly, this minimized the presence of smaller unrelated peptides in the sample, and also excluded precipitate (typically undigested proteins) and polymers that may elute from the C8 resin when using very high (>50%) concentrations of acetonitrile.

For our SUMO-IP strategy, desalted Lys-C-digested peptides were lyophilized and re-dissolved in a very mild buffer. In this manner, the SUMO purification was carried out in buffer conditions analogous to western blotting, where the 8A2 antibody is known to recognize the SUMO epitope in a robust and specific manner. To corroborate this, depletion of SUMOylated peptides upon enrichment from a Lys-C digested lysate was confirmed by immunoblot (IB) analysis (Supplementary Fig. 2A-B). Compared to a previous study where the 8A2 antibody is used to immunoprecipitate endogenously SUMOylated proteins, we used 20 times less antibody per milligram of starting material[3], while achieving a 20-fold greater depth of sequencing (Fig. 1E).

Choosing the optimal SUMO mass remnant

While successful in enriching peptides modified by endogenous SUMO2/3, the single-largest complication of proteomics analysis of endogenous site-specific SUMO2/3 relates to the large mass remnant that remains after Lys-C or trypsin digestion. Thus, knowing that a Lys-C digestion followed by purification and a second enzymatic digestion can generate samples of sufficient purity for proteomics analysis[1,4], we generated initial SUMO-IP samples from HeLa and U2OS cells (Supplementary Fig. 2C), and digested equal amounts with either trypsin, endoproteinase Glu-C (cleaves C-terminal of E and D residues), and wildtype alpha-lytic protease (WALP; preferentially cleaves C-terminal of V, A, T, and S residues). The tryptic digest was expected to leave a 3.5 kDa SUMO remnant, while Glu-C digest theoretically leaves a 1.3 kDa remnant, and WALP should yield a di-glycine remnant.

For identification of endogenous SUMO modification sites we employed liquid chromatography coupled with high-resolution mass spectrometry (LC-MS/MS) on the Q Exactive

HF instrument[5]. For identification of SUMO-modified lysine residues and associated SUMO mass remnants, all peptides were fragmented using higher-energy collisional dissociation (HCD)[6]. To ensure high confidence in identified peptides and modification sites, acquired data was filtered at both peptide- and protein-level to ensure a false-discovery rate (FDR) or <1%. For identification of modified lysines we assessed the localization score for each site using the MaxQuant software suite[7], and only considered sites identified with an Andromeda score above 40, a delta score above 20, and a localization score above 6 (approximately >80% localization probability).

Although the tryptic SUMO mass remnant is commonly considered too cumbersome for successful identification, we nonetheless identified 918 unique SUMOylated lysines using HCD fragmentation (Supplementary Fig. 2D and Supplementary Data 1). Using Glu-C, we identified 1,313 unique sites, although the MS analysis was hampered by the fact that Glu-C digestion resulted in four distinct mass remnants because of variable cleavage of the SUMO2/3 C-terminus (Supplementary Data 1). Using WALP, we identified 1,144 unique SUMO2/3 sites, with identification hampered because digestion of proteins with both Lys-C and WALP frequently generated peptides too short for reliable sequencing by MS/MS. Moreover, we observed that WALP exhibits a very wide and variable preference for peptide backbone cleavages, leading to a large theoretical peptide database and accordingly low scores after applying FDR. Nonetheless, we identified >2,500 unique SUMO sites in this initial pilot (Supplementary Fig. 2D and Supplementary Data 1), with relatively few sites identified by multiple enzymes.

Based on these initial observations, we performed a second comparison, where endoproteinase Glu-C was substituted with endoproteinase Asp-N. We reasoned that Asp-N could provide a more consistent generation of a singular mass remnant as the most C-proximal acidic residue in SUMO2/3 is an aspartic acid. As previously, we performed one set of SUMO-IP on HeLa and U2OS cells, and split the samples three-way to facilitate comparison between the enzymes (trypsin, Asp-N and WALP). In this second comparison we identified 749 sites with trypsin, 626 sites with WALP, and a striking 3,030 sites with Asp-N (Supplementary Fig. 2D and Supplementary Data 1). Reassuringly, digestion with Asp-N of the SUMO C-terminus resulted in a single mass remnant, encompassing ~99.9% of all signal. Moreover, at 960 Da, the mass remnant could be consistently resolved and generated a plethora of diagnostic mass fragments in the MS/MS spectra (Supplementary Data 13). Overall, when normalizing the number of sites identified (Supplementary Fig. 2E), Asp-N proved 4-fold more effective than the other three enzymes.

In conclusion, while optimizing the SUMO site identification strategy, we utilized four different enzymes in the second stage, all following Lys-C as the first stage enzyme. Comparatively, we found that Asp-N identified four times more sites as WALP and trypsin did, when using equal amounts of sample. Unlike Glu-C, Asp-N did not generate a multitude of mass

remnants when digesting the SUMO2/3 C-terminus. Somewhat to our surprise, the tryptic remnant could readily be identified using our approach in combination with the freely available MaxQuant software[7,8], although efficient separation of precursors by liquid chromatography presented the largest technical challenge as hydrophobic properties of peptides were dominated by the large SUMO mass remnant. On the other hand, WALP digestion combined with Lys-C resulted in a large amount of precursors that were too small to be identifiable, suggesting that WALP treatment leads to over-cleavage of peptides, and moreover WALP appeared to display a considerably greater amount of non-specific cleavage as compared to the other enzymes tested. Nonetheless, both trypsin and WALP displayed unique cleavage patterns, and are fully compatible with our method, and could thus be considered as complementary digestion strategies in order to maximize breadth of sequencing. More importantly, identifying SUMO sites through multiple enzymatic approaches is a good way to minimize potential false-discovery.

SUPPLEMENTARY NOTE 2

Mass spectrometric analysis strategy for all main experiments

For all main SUMO-IP MS experiments based upon Lys-C and Asp-N double digestion, final purified peptide samples were pre-fractionated into six fractions before MS analysis using high-pH fractionation on C18 StageTips, essentially as previously described[1]. Each fraction was analyzed using 80 minutes of LC-MS/MS gradient time including a column washing block, hereby allowing an endogenous SUMOylome to be analyzed in eight hours of mass spectrometric acquisition time.

For identification of endogenous SUMO2/3 modification sites we employed nanoscale liquid chromatography coupled with high-resolution tandem mass spectrometry (LC-MS/MS) on the Q-Exactive HF instrument[5]. For identification of SUMO-modified lysine residues and associated SUMO2/3 mass remnants, all peptides were fragmented using higher-energy collisional dissociation (HCD)[6]. All MS raw data was processed using the MaxQuant software suite[7], and the obtained data was stringently filtered at the peptide-spectrum-match, protein, and modified-site levels. The ~1.0 kDa mass remnant resulting from Asp-N digestion of SUMO2/3 generated a unique range of diagnostic MS/MS fragments, which were further utilized to increase SUMO2/3 site identification confidence (Supplementary Data 13).

SUPPLEMENTARY NOTE 3

Comparison of the identified endogenous SUMOylome to exogenous analyses

As our endogenous analysis was performed using the same treatments as our recent K0-SUMO study, we investigated in more detail the differences in identified SUMOylation sites between the endogenous and exogenous analyses. Under control conditions and in response to heat shock we identified 70% and 86% of the number of previously identified exogenous SUMO sites, respectively (Fig. 1D). However, while the K0-SUMO experiments were carried out at a similar scale, they were performed in HeLa and U2OS cells, whereas we used HEK cells for our in-depth endogenous analysis. Interestingly, we did not identify nearly as many endogenous SUMO2/3 sites in response to MG132, although any direct comparison here is obfuscated by the different cell line used. Still, when compared to a recent exogenous SUMO2/3 proteomics screen using MG132 in HEK cells[9], we identified 82% of the number of previously identified exogenous SUMO sites.

Within our endogenous data, total accumulation of MS/MS spectral intensity corresponding to SUMO sites in response to heat shock doubled over the course of one hour, while only a 40% increase in total SUMO site intensity was detected over 8 hours treatment of MG132 (Supplementary Data 1). Corroborating this, quantification of internal peptides correlating to total SUMO-2/3 purified after Lys-C digest demonstrated a 55% increase after heat shock, and a 25% increase after MG132 treatment (Supplementary Fig. 3D).

SUPPLEMENTARY NOTE 4

Analysis of SUMO consensus sub-motifs and sites-per-protein

When investigating the sequence context surrounding the endogenously SUMOylated lysine residues we identified, other known sequence trends could be observed (Fig. 2A), including enrichment for bulky hydrophobic amino acids at −1, acidic residues from +5 to +8, and the inverted [ED]xK motif[10]. To further validate the motifs observed within our data, we performed Motif-X analysis to extract high-confidence sequence motifs (Supplementary Data 1 and Supplementary Fig. 4), confirming the presence of all well-established SUMO motifs within our dataset. KxE-type sites were globally more abundantly modified and thus more confidently identified, and the top 2,000 sites identified retained over 50% KxE adherence (Fig. 2B), demonstrating that identified SUMO2/3 sites and the overall SUMOylation pattern identified by our method follows the previously described preferences of the SUMO enzymatic machinery.

Since our endogenous method identified sites following known preferences of SUMOylation, we investigated the number of SUMO sites identified per protein. Our endogenous analysis identified ~3 SUMOylation sites per protein under standard growth conditions and in response to MG132, and ~4 sites per protein in response to heat shock (Fig. 2C-D). In response to stress, we observed a sharper incline in the number of SUMO-modified lysines compared to SUMO target proteins (Fig. 2C-D), suggesting that primarily the same proteins are being increasingly modified in response to proteotoxic stress and heat shock, in line with previous SUMO proteomics reports[1,4].

SUPPLEMENTARY NOTE 5

Comparison between SUMOylation phenomena in human and mouse

We compared identified SUMO2/3 target proteins, grouped by their respective protein-coding genes, in order to approximate global similarities and differences between the SUMOylomes we identified from cultured human cells and mouse organs. Overall, 663 proteins were found to be SUMOylated in both species, equaling 69.4% of all SUMO2/3 target proteins we identified in mouse (Supplementary Data 9). When only considering human proteins found to be SUMOylated under standard growth conditions, the overlap was 551 proteins. To further elaborate this, we performed sequence homology comparison between SUMO2/3 target proteins identified in human and mouse, and found that 46.1% of proteins were modified on at least one conserved lysine residue. At the site level, 41.4% of overlapping SUMOylation events occurred on conserved lysine residues. SUMO2/3 target protein scores globally correlated between human and mouse with R=0.50 (Supplementary Data 9).

To highlight global SUMOylation differences between HEK cells and mouse organs, we used annotation enrichment analysis to look for enriched terms in clusters of SUMO2/3 targets (Supplementary Data 10). Proteins SUMOylated in both model systems were found to be enriched in canonical functions of SUMOylation, such as modification of the SUMO machinery itself, transcriptional regulation, and chromatin remodeling – highlighting the importance of SUMOylation in these processes regardless of cell state. When looking at SUMOylation unique to HEK cells, we observed enrichment for proteins involved in telomere maintenance, response to ionizing radiation, helicases, and rRNA processing. In mouse organs, exclusively SUMOylated proteins were strongly enriched for many functions associated with organs, including glycolysis, oxygen transport, and muscle contraction. SUMO2/3 target proteins found in cell culture in response to stress exclusively were primarily enriched for tRNA processing, the Golgi apparatus, and both phosphatase and kinase function. Interestingly, 112 proteins only SUMOylated in response to stress in cell culture were baseline SUMOylated in mouse organs, with functions including regulation of muscle adaptation to stress, oxidation-reduction activity, and nutrient response, suggesting that the conditions required for this stress-induced SUMOylation in cell culture may exist naturally within certain mouse tissues.

Subcellular localization of endogenous SUMO substrates

SUMOylation is canonically a modifier of nuclear proteins[11], and proteomics studies have extensively supported this[12]. In order to assess whether endogenous and *in vivo* SUMOylation has a similar tendency for targeting nuclear proteins, we investigated the subcellular localization

annotation for all SUMO2/3 proteins and sites identified. In the human cell line data, 76% of all SUMO-modified proteins were localized in the nucleus, increasing to 88% when considering SUMO sites individually (Fig. 5A). Under control condition, 95% of SUMO sites occurred on nuclear proteins, confirming that steady-state endogenous SUMOylation is predominantly nuclear. Moreover, 29% of SUMO sites were mapped to proteins located at the chromatin; 5-fold greater than randomly expected. We performed the same analysis for the mouse organs, and found a large degree of similarity to human cell culture (Fig. 5B). Of all SUMO-modified proteins in mouse organs, 79% were nuclear-localized and 20% chromatin-localized, with no notable differences when considering SUMO target proteins or sites. Lung and spleen displayed the highest fraction of nuclear-localized SUMO target proteins, at 94% and 96%, respectively. Testis and spleen displayed the highest fraction of chromatin-localized SUMO sites, at 29%.

Crosstalk between phosphorylation and SUMOylation

Phosphorylation at the +4 or +5 position of the SUMOylated lysine has been previously established, as well as a tendency for this phosphorylation to be proline-directed[1]. Using our endogenous SUMO2/3 method in HEK cells, we mapped 526 unique SUMO2/3 and phosphorylation co-modified peptides, directly identifying and localizing both PTMs in the MS/MS spectra (Supplementary Data 11). Proline-directed phosphorylation was observed in 70% of all cases, and thus in agreement with what was described previously. In humans, proline-directed phosphorylation occurs with 34.7% frequency[13], highlighting the significance of our finding with a *p*-value of $2.5*10^{-23}$ using Fisher Exact testing. An enrichment for phosphorylation at the +4 and +5 positions was also confirmed (Fig. 5C), however, we also observed significantly more phosphorylation events at both the −3 and +2 positions and significantly less phosphorylation events at both the −1 and +1 positions. Overall, we found 3.5% of total SUMOylation proximal to phosphorylation under standard growth conditions, and 2.7% when also considering proteotoxic stress.

In mouse organs, we identified 49 unique SUMO2/3 and phosphorylation co-modified peptides in total (Supplementary Data 12). Phosphorylation occurred significantly at +5 in relation to the SUMOylated lysine (Fig. 5D), in accordance with the canonical phosphorylation-dependent SUMOylation motif[14]. We also observed higher occurrence of phosphorylation at −5, −4, and −3, of SUMOylation. Notably, we could not detect any co-modified peptides in brain and heart, and the overall contribution of SUMO-phospho to the total SUMOylome was only ~0.1% in muscle, lung, and kidney. In spleen, 0.7% of SUMOylation occurred proximal to phosphorylation, increasing further to 1.5% for both liver and testis. Globally, 73% of SUMO-proximal phosphorylated occurred in a proline-directed manner, suggesting that phosphorylation that occurred proximal to SUMO in

organs is potentially cell-cycle regulatory. In mice, proline-directed phosphorylation occurs with 37.5% frequency[15], showing the significance of our finding with a *p*-value of 0.0022 using Fisher Exact testing. Interestingly, 76% of all co-modified peptides could be identified in liver, with the rest mapped primarily in testis, and otherwise in spleen. In testis, ~90% of the total phosphorylation-proximal SUMOylation occurred on Mediator of DNA damage checkpoint 1 (Mdc1). Although Mdc1 is expressed at a somewhat higher level in testis as compared to the other organ types we investigated[16], Mdc1 was nonetheless found to be co-modified to a higher degree than the canonical phosphorylation-dependent SUMOylation target protein Nop58[17]. Conversely, Nop58 was SUMO-phospho co-modified to a higher degree in other organ types.

Structural preference of endogenous SUMOylation

Exogenous SUMOylation generally targets lysines residing in disordered and exposed protein regions, contrasting ubiquitylation which frequently targets lysines in globular and buried protein regions[1]. To investigate whether the same preference is observed for endogenous SUMOylation in human and mouse, we performed structural-predictive analyses on all human and mouse SUMO2/3 target proteins identified. In human cells, we observed that SUMO2/3 sites identified under standard growth conditions were 60% more likely to occur in disordered regions (Fig. 6A). In response to heat shock, there was still enrichment for SUMOylation in disordered regions, but notably less than in the control. In response to MG132, and especially when considering MG132-exclusive SUMO sites, the structural preference of SUMOylation was completely inverted, essentially mimicking ubiquitylation. In mouse organs, we observed a global preference for SUMOylation to target disordered regions, positioned in between the control and stress-induced enrichments observed for human cells (Fig. 6B). When considering specific organs, we noted that in brain there was no significant enrichment for any structural regions, in contrast to all other organs. Conversely, SUMOylation in lung and spleen was more likely to be targeted to disordered regions as compared to other organ types.

To assess whether these slight differences in structural preferences are owing to SUMO, or to a more general aspect of PTMs in tissue, we mapped tissue-specific ubiquitylation sites onto the SUMO2/3 proteins identified across organs. Collectively, the structural preferences of ubiquitin were highly similar across tissues and in human cells. Lysines targeted by both PTMs in mouse organs did not display a notable type of structural preference. To further disentangle potential organ-specific differences, we additionally compared SUMO to ubiquitin in individual organs (Fig. 6C); however, only for the five organs where ubiquitylation was previously mapped[18]. Here, we observed the same trend, with SUMO targeting disordered regions in heart, kidney, liver, and muscle, whereas ubiquitin predominantly targeted structured regions in all five organs, and most

notably in liver. Overall, this demonstrates the intriguing propensity for SUMO2/3 and ubiquitin to target different structural regions of the same proteins. With these preferences appearing to be globally different between organ types, this highlights the need for future investigation into the *in vivo* regulation of these PTMs.

SUPPLEMENTARY NOTE 6

SUMO density in cells and organs

To assess the purity of our SUMO-IP strategy, we quantified the total amount of SUMO2/3-modified peptides and proteins relative to the background signal. In HEK cells between 93-96% of MS/MS-identified peptide signal originated from SUMOylated proteins, corresponding to the overall sample purity after SUMO-IP (Supplementary Fig. 9A), and demonstrating the remarkable efficiency of our purification strategy. In mouse organs, the SUMO-IP purity ranged from 42-62%, indicating either a larger relative presence of background proteins, or more putative target proteins where we did not manage to identify a SUMO site. Because the samples were further digested after the SUMO-IP, not all identified peptides correspond to modified peptides, and in HEK cells between 34-50% of SUMO target protein signal was derived from SUMO sites. In mouse, this number was considerably lower, ranging from 3-4% in muscle, heart, and brain, to 16-22% in spleen and testis. As modified peptides were harder to identify and filtered more stringently than unmodified peptides, the observed peptide-level purity correlated closely to the overall number of SUMO sites identified across the samples.

By normalizing the absolute amount of SUMO protein purified to the amount of input material used for each experiment, we observed a much greater yield of SUMO target proteins per milligram of starting material in HEK cells, as compared to the mouse organs (Supplementary Fig. 9B). Even under standard growth conditions, we found a >10-fold higher density of SUMO-modified proteins in HEK cells, increasing to >20-fold in response to heat shock. No large differences were observed between organ types, with only heart having a lower yield (Supplementary Fig. 9C). At the SUMO sites level, the gap was considerably bigger, with control condition HEK cells having a 30-fold higher yield of SUMO sites as liver, increasing to a 70-fold higher yield in response to heat shock. At the SUMO sites level, some differences were observed between the organs, mostly mirroring the sample purity as outlined above.

Finally, to ascertain whether the decreased SUMO yield obtained from mouse organs could be because of technical limitations, we investigated the amount of background proteins identified across all experiments, in relation to the amount of starting material (Supplementary Fig. 9D). Reassuringly, even though we observed very large differences in the amount of SUMO sites and proteins between the experiment types, the amount of normalized absolute background signal did not vary significantly between samples. This suggested that the decreased amount of SUMO observed in mouse organs is because of biological reasons.

SUPPLEMENTARY NOTE 7

Validation of SUMO equilibrium using immunoblot analyses

Whereas immunoblotting can be used to a certain extent to assess the distribution of conjugated and free SUMO, differences in blotting methods and antibody preferences can bias towards detection of either conjugated SUMO or free SUMO, and such methods are not always applicable across all vertebrate model systems. Nonetheless, to validate the large difference between SUMO equilibrium we observed when comparing HEK cells to mouse organs, we performed immunoblot analysis on whole organ extracts and HEK whole cell lysates (Supplementary Fig. 11). To facilitate immunoblot analysis and prevent potential deconjugation of SUMO2/3 during sample preparation, these samples were prepared in a denaturing buffer including a high concentration of sodium dodecyl sulfate, broad-range protease inhibitors, and a high concentration of N-ethylmaleimide to specifically inhibit SUMO proteases[2].

In agreement with our MS data, we observed a much higher density of SUMOylation in HEK cells compared to all mouse organs we tested, exemplified by similar or higher levels of SUMO2/3 signal observed in HEK cells despite loading 10 times less total protein (Supplementary Fig. 11). Reassuringly, we also observed comparable distributions of conjugated SUMO2/3 and free SUMO2/3, with SUMO2/3 predominantly conjugated in HEK cells and large pools of free SUMO2/3 existing natively in most mouse organs. Although immunoblot analysis can be used to probe the SUMO equilibrium, potential differences in transfer efficiency of small and large proteins during electrophoretic transfer, in addition to potential off-target effects when using different antibodies to probe for SUMO2/3 in distinct organ types, could lead to variable results. As our MS strategy is not sensitive to these limitations, we believe that using MS to quantify the SUMO equilibrium is more accurate and reproducible.

SUPPLEMENTARY NOTE 8

Comparison to contemporary endogenous SUMO proteomics methods

Whereas other SUMO proteomics strategies have been published that facilitate studying endogenous SUMOylation to some degree[3,19-21], none of them display the same sensitivity as our method, and none of them can specifically enrich and uniquely identify lysines modified by endogenous SUMO-2/3. Two contemporary methods purify SUMOylated proteins, but do not strife to identify modified lysine residues[3,19].

Comparison to site-specific endogenous SUMO strategies

Recently, a study was published that facilitates identification of endogenously SUMOylated lysines, through digestion of total lysate with the WALP enzyme followed by di-glycine enrichment[21]. In contrast to the workflow we used, Lumpkin et al. did not perform pre-purification for SUMO2/3, and relied solely on the WALP enzyme to preferentially cleave C-terminal of the threonine residue in the SUMO C-terminus, thereby revealing the di-glycine residue for IP. Thus, the method described by Lumpkin et al. is unable to distinguish SUMO-1 from SUMO-2/3, as their strategy does not generate a mass remnant unique to any SUMO. Moreover, Lumpkin et al. point out that WALP is a promiscuous enzyme which can cleave C-terminal of virtually any amino acid, including C-terminal of arginine[21]. This in turn releases the di-glycine remnant from ubiquitin, allowing ubiquitin sites to be purified and misinterpreted as SUMO sites. The authors further note that di-glycine remnants originating from other ubiquitin-like proteins could also be misidentified using their method[21]. We integrated the data from Lumpkin et al. into our comparison of SUMO proteomics screens, and observed poor adherence to the KxE consensus, poor overlap to other SUMO proteomics studies, and high overlap to ubiquitin proteomics studies, thereby suggesting a high probability of the method described by Lumpkin et al. false-positively identifying ubiquitin sites as SUMO sites (Supplementary Data 3 and 4).

Another recent study identified 53 lysines endogenously modified by SUMO1 in mouse testis, applying a strategy of trypsin/Lys-C digest followed by SUMO1 peptide purification and subsequent digestion with Glu-C[20]. Although not reaching the same depth as our method and not being applicable to SUMO2/3, the method described by Cai et al. is the only one to currently facilitate specific study of lysine residues endogenously modified by SUMO1.

In-depth analysis of endogenous SUMO sites identified by Lumpkin et al.

The highest number of reported endogenous SUMO sites detected by mass spectrometry is 1,209, with the authors using heat shock treated HeLa cells, digesting the total lysate with the WALP enzyme, and subsequently performing di-glycine enrichment to purify putative SUMO sites[21]. In theory, with WALP cleaving C-terminal of threonine, this would allow identification of lysines modified by endogenous SUMO-1 and SUMO-2/3, although these distinctly regulated SUMO family members cannot be distinguished by their method. In our initial optimization phase, we also tested WALP, and noticed that the enzyme is highly promiscuous. While WALP preferentially cleaves C-terminal of valine, alanine, threonine, serine, leucine, and glycine residues, the enzyme can cleave virtually anywhere at somewhat lower efficiency. This is reflected by Lumpkin et al. performing a non-specific search on their data, allowing WALP to cleave anywhere for generation of their theoretical peptide library. Moreover, the authors found multiple SUMOylated peptides that were cleaved C-terminal of arginine, and mention that up to 8% of their peptides were cleaved C-terminal of arginines[21]. This in turn strongly suggests that ubiquitin, which has an RGG motif at the C-terminus, could be cleaved by WALP and subsequently falsely identified as SUMOylation after di-glycine purification. Lumpkin et al. present data utilizing SUMO-specific proteases as a control, and observed 12% of their putative SUMO sites did not notably decrease on SUMO protease treatment, suggesting that the false-discovery rate of their method is at least 12%. Whereas for identification of abundant SUMO sites this may not be a major issue, the probability of false discovery of ubiquitin sites as SUMO sites would increase when sequencing samples to a greater depth, or when studying dynamic responses.

Because during our WALP pilot experiments we first purified peptides modified by SUMO-2/3 following Lys-C digestion, we largely negate the potential misidentification of SUMO-2/3 sites owing to di-glycine remnants resulting from digestion of either SUMO-1, ubiquitin, or other ubiquitin-like modifiers. Moreover, our main strategy depends on the use of LysC in combination with AspN, which leaves a short peptide that uniquely identifies SUMO-2/3 (Supplementary Data 13), thereby entirely circumventing the use of a non-unique mass remnant.

Since we performed a thorough comparison between all major SUMO proteomics studies published to date (Supplementary Data 3), we utilized this insight to more closely evaluate the data reported by Lumpkin et al. Out of the 1,192 SUMO sites that we could map to current human Uniprot identifiers, 32.6% adhered to the canonical KxE consensus motif. Notably, SUMO proteomics studies identifying similar numbers of sites in cell lines generally identify considerably higher adherences, including 49.5% out of 1,042 sites under standard growth conditions[22], 47.5% out of 1,001 sites in response to heat shock[23], and 44.1% out of 953 sites in response to MG132 treatment[24]. The top 10 percent of sites identified in our study matched 54.6% KxE consensus for

1,486 sites. In our WALP pilot data, we identified a total of 1,293 SUMO sites using WALP following Lys-C digest and SUMO-2/3 IP, of which 47.8% matched KxE consensus, in line with expectations. Thus, the adherence of the SUMO sites identified by Lumpkin et al. is notably lower than would be expected, suggesting ubiquitin sites being identified, as these do not adhere to any consensus motif.

Further, 36.5% of the SUMO sites identified by Lumpkin et al. were not previously identified by any other SUMO proteomics study. The amount of non-overlap was only 2.0%, 3.3%, and 7.2% for three other studies of similar size[22-24], with all three methods using different purification strategies and digestion patterns. In our study, the amount of non-overlap was only 11.8% for the top 1,486 of sites identified, confirming that our sites conform to commonly identified SUMO sites, despite our method being endogenous and using an alternative digestion pattern. From our WALP pilot data, 21.3% of sites were not found in other screens, and matched 25.1% KxE. The 435 non-overlapping SUMO sites identified by Lumpkin et al. matched only 12.9% KxE consensus, whereas all our 4,910 non-overlapping sites still retained 16.5% KxE despite a >10-fold greater depth of sequencing. Taken together, this demonstrates a relatively poor overlap between sites identified by Lumpkin et al. and previously published SUMOylation sites.

Finally, as some lysine residues may be modified by either SUMO or ubiquitin, we investigated the average overlap between these modifications based on published proteomics data (Supplementary Data 3). On average, 15,535 SUMOylated lysines detected by at least 2 SUMO screens were also detected as ubiquitylated in ~8.3 screens. In our data, 14,869 SUMOylated lysines were also detected as ubiquitylated in ~7.6 screens, and the 1,293 SUMOylated lysines detected in our WALP pilot data were also detected as ubiquitylated in ~8.7 screens, closely conforming to the published standard. In the data reported by Lumpkin et al., 1,192 SUMOylated lysines were also detected as ubiquitylated in ~18.4 screens. The fact that the SUMO sites identified by Lumpkin et al. were more than twice as frequently identified as ubiquitylated moreover suggests a high rate of false-positive identification.

SUPPLEMENTARY NOTE 9

Evaluation of the efficiency of our strategy compared to other endogenous methods

To demonstrate that the analytical superiority of our method compared to other recently published endogenous methods does not simply stem from the fact we utilized a considerable amount of mass spectrometric machine time, we investigated the number of SUMO2/3 sites directly detected by MS/MS in single replicate fractions, corresponding to only 60 minutes of machine time (Supplementary Fig. 13A). In HEK cells, we routinely identified 1,500 SUMO2/3 sites per fraction under standard growth conditions and 4,000 SUMO2/3 sites per fraction in response to heat shock, thereby outclassing the number reported by Lumpkin et al. nearly 4-fold with only 60 minutes of machine time while using comparable equipment. Similarly, in the considerably more technically challenging setting of mouse organs, we identified up to 300 SUMO2/3 sites in single mouse liver fractions, and up to 200 SUMO2/3 sites in single mouse testis fractions (Supplementary Fig. 13B), outnumbering all sites reported by Cai et al. 4-fold while using just 60 minutes of MS machine time and comparable equipment. Moreover, even though we did not perform single-shot measurements, fractionation is an optional step of our strategy and leaving out fractionation would likely further increase identification density while requiring less machine time[1], although the maximum depth of sequencing would be limited. The primary reason for dedicating significant amounts of machine time towards the measurement of the SUMOylomes contained in this manuscript was to provide quantitative accuracy and reproducibility, and to assemble a comprehensive and valuable resource of *in vivo* SUMOylation sites.

SUPPLEMENTARY NOTE 10

Validation of matching between runs

We investigated the intensity assignments for MS/MS and matching identifications within all the mouse organ SUMO data. For this, we utilized the "evidence.txt" file as written by MaxQuant, which is available at the ProteomeXchange Consortium database via the Proteomics Identifications (PRIDE) partner repository, under dataset ID PXD008003. Overall, we found that 16.7% of the total assigned SUMO signal was derived from matching between runs, with the large majority of all experimental evidence derived from direct MS/MS identifications. Upon inspection of all matched peptides, we found that 79.5% of these occurred within replicates of the same organ type, and 96.5% matched to the same chromatographic fraction. The 3.5% of matches corresponding to neighboring fractions could potentially be false-positive, which would correspond to 0.6% of the total experimental intensity, leaving the maximum matching FDR below 1%. Moreover, fraction-mismatched hits are not necessarily wrong as peptides frequently appear in neighboring chromatographic fractions.

SUPPLEMENTARY DISCUSSION

Evolutionary conservation of SUMO

We investigated the evolutionary conservation of SUMOylation, and found that SUMOylation occurred on less conserved lysine residues in ordered regions. In support of our findings, bioinformatics analysis of PTM co-evolution previously revealed that SUMOylation entailed the fastest evolution of all analyzed PTMs[25]. Contrary to what we found, Minguez et al. reported that SUMOylation sites were more conserved compared to background residues. However, the data used by Minguez et al. for this analysis was based upon a limited number of SUMOylation sites, mostly derived from studies employing single mutagenesis analyses, which overall does not reflect an unbiased overview of the SUMOylome. Intriguingly, whereas SUMO2/3 targeted evolutionarily less conserved lysines, most PTMs are described to target more conserved residues[25], and specifically phosphorylation is considered to occur on evolutionarily conserved residues[26]. Thus, our data highlights a unique property of SUMOylation to rapidly evolve, suggesting that SUMOylation may be regulated or potentially misregulated to a considerable extent in the context of disease.

Endogenous and *in vivo* SUMO chain topology

SUMO2/3 chain topology was similarly different between cell culture and organs, with SUMO chains being focused on the KxE-type K11 in SUMO2, and increasingly so in response to stress. Conversely, K11 modification of SUMO2 was rarer in organs, with chain formation more often occurring on K21 and K33. Interestingly, this would shift the chain topology observed in organs in the opposite direction from the stress-induced topology in cell lines, thus hinting at a ‘low stress’ state in organs. In a recent study using the lysine-deficient K0-SUMO, nearly 90% of K0-SUMO modification occurred on K11 in endogenous SUMO2, with almost no modification on the other lysines[1]. Since the K0-SUMO would effectively prevent a chain from being extended, this suggests that K11 may be the first linkage formed in a branched chain, or alternatively that overexpressed or mutated SUMO is more likely to be targeted to K11, or less likely to be removed from K11. With no SUMO E3 ligases known to facilitate specific types of SUMO chain linkages, it otherwise remains unknown how these observed differences in chain topology may have arisen. However, with the amount of SUMO residing in chains being globally relatively low, SUMO chains may only have niche functions in vertebrate cells and organs. With two SUMO-specific proteases specifically targeted at reducing SUMO chains[27], it may be possible that SUMO is primarily intended to laterally SUMOylate multiple lysines in proteins, as opposed to vertically forming SUMO chains on singular lysines. In turn, this could explain the large degree of overlap observed between

SUMOylated lysines identified using either the endogenous method described here, or using the lysine-deficient K0-SUMO[1,4].

SUMOylation patterns across distinct organ types

Our findings demonstrate overlapping SUMOylation patterns between certain organ types, and suggest that shared biological processes may be commonly governed by SUMOylation. Specifically, heart and skeletal muscle were enriched for multiple overlapping functions, which is logical considering both are a type of muscle. Moreover, similar SUMOylation patterns observed in liver and kidney are cooperative for certain biological regulations through tight regulation of neural and humoral mechanisms, while similarity of the SUMOylome across spleen and lung may reflect commonly shared immune activities between splenic lymphocytes and airway epithelia. In support of this, SUMO is generally known to regulate the immune system, and be involved in auto-immune diseases[28].

SUPPLEMENTARY REFERENCES

1. Hendriks, I. A., Lyon, D., Young, C., Jensen, L. J., Vertegaal, A. C., & Nielsen, M. L. Site-specific mapping of the human SUMO proteome reveals co-modification with phosphorylation. *Nat. Struct. Mol. Biol.* **24**, 325-336 (2017).

2. Barysch, S. V., Dittner, C., Flotho, A., Becker, J., & Melchior, F. Identification and analysis of endogenous SUMO1 and SUMO2/3 targets in mammalian cells and tissues using monoclonal antibodies. *Nat. Protoc.* **9**, 896-909 (2014).

3. Becker, J., Barysch, S. V., Karaca, S., Dittner, C., Hsiao, H. H., Berriel, D. M., Herzig, S., Urlaub, H., & Melchior, F. Detecting endogenous SUMO targets in mammalian cells and tissues. *Nat. Struct. Mol. Biol.* **20**, 525-531 (2013).

4. Hendriks, I. A., D'Souza, R. C., Yang, B., Verlaan-de Vries, M., Mann, M., & Vertegaal, A. C. Uncovering global SUMOylation signaling networks in a site-specific manner. *Nat. Struct. Mol. Biol.* **21**, 927-936 (2014).

5. Scheltema, R. A., Hauschild, J. P., Lange, O., Hornburg, D., Denisov, E., Damoc, E., Kuehn, A., Makarov, A., & Mann, M. The Q Exactive HF, a Benchtop mass spectrometer with a pre-filter, high-performance quadrupole and an ultra-high-field Orbitrap analyzer. *Mol. Cell Proteomics.* **13**, 3698-3708 (2014).

6. Olsen, J. V., Macek, B., Lange, O., Makarov, A., Horning, S., & Mann, M. Higher-energy C-trap dissociation for peptide modification analysis. *Nat. Methods* **4**, 709-712 (2007).

7. Cox, J. & Mann, M. MaxQuant enables high peptide identification rates, individualized p.p.b.-range mass accuracies and proteome-wide protein quantification. *Nat. Biotechnol.* **26**, 1367-1372 (2008).

8. Cox, J., Neuhauser, N., Michalski, A., Scheltema, R. A., Olsen, J. V., & Mann, M. Andromeda: a peptide search engine integrated into the MaxQuant environment. *J. Proteome. Res.* **10**, 1794-1805 (2011).

9. Lamoliatte, F., McManus, F. P., Maarifi, G., Chelbi-Alix, M. K., & Thibault, P. Uncovering the SUMOylation and ubiquitylation crosstalk in human cells using sequential peptide immunopurification. *Nat. Commun.* **8**, 14109 (2017).

10. Matic, I., Schimmel, J., Hendriks, I. A., van Santen, M. A., van de Rijke, F., van Dam, H., Gnad, F., Mann, M., & Vertegaal, A. C. Site-specific identification of SUMO-2 targets in cells reveals an inverted SUMOylation motif and a hydrophobic cluster SUMOylation motif. *Mol. Cell* **39**, 641-652 (2010).

11. Rodriguez, M. S., Dargemont, C., & Hay, R. T. SUMO-1 conjugation in vivo requires both a consensus modification motif and nuclear targeting. *J. Biol. Chem.* **276**, 12654-12659 (2001).

12. Hendriks, I. A. & Vertegaal, A. C. A comprehensive compilation of SUMO proteomics. *Nat. Rev. Mol. Cell Biol.*(2016).

13. Hornbeck, P. V., Kornhauser, J. M., Tkachev, S., Zhang, B., Skrzypek, E., Murray, B., Latham, V., & Sullivan, M. PhosphoSitePlus: a comprehensive resource for investigating the structure and function of experimentally determined post-translational modifications in man and mouse. *Nucleic Acids Res.* **40**, D261-D270 (2012).

14. Hietakangas, V., Anckar, J., Blomster, H. A., Fujimoto, M., Palvimo, J. J., Nakai, A., & Sistonen, L. PDSM, a motif for phosphorylation-dependent SUMO modification. *Proc. Natl. Acad. Sci. U. S. A* **103**, 45-50 (2006).

15. Hornbeck, P. V., Zhang, B., Murray, B., Kornhauser, J. M., Latham, V., & Skrzypek, E. PhosphoSitePlus, 2014: mutations, PTMs and recalibrations. *Nucleic Acids Res.* **43**, D512-D520 (2015).

16. Santos, A., Tsafou, K., Stolte, C., Pletscher-Frankild, S., O'Donoghue, S. I., & Jensen, L. J. Comprehensive comparison of large-scale tissue expression datasets. *PeerJ.* **3**, e1054 (2015).

17. Westman, B. J. & Lamond, A. I. A role for SUMOylation in snoRNP biogenesis revealed by quantitative proteomics. *Nucleus.* **2**, 30-37 (2011).

18. Wagner, S. A., Beli, P., Weinert, B. T., Scholz, C., Kelstrup, C. D., Young, C., Nielsen, M. L., Olsen, J. V., Brakebusch, C., & Choudhary, C. Proteomic analyses reveal divergent ubiquitylation site patterns in murine tissues. *Mol. Cell Proteomics.* **11**, 1578-1585 (2012).

19. Bruderer, R., Tatham, M. H., Plechanovova, A., Matic, I., Garg, A. K., & Hay, R. T. Purification and identification of endogenous polySUMO conjugates. *EMBO Rep.* **12**, 142-148 (2011).

20. Cai, L., Tu, J., Song, L., Gao, Z., Li, K., Wang, Y., Liu, Y., Zhong, F., Ge, R., Qin, J., Ding, C., & He, F. Proteome-wide Mapping of Endogenous SUMOylation Sites in Mouse Testis. *Mol. Cell Proteomics.* **16**, 717-727 (2017).

21. Lumpkin, R. J., Gu, H., Zhu, Y., Leonard, M., Ahmad, A. S., Clauser, K. R., Meyer, J. G., Bennett, E. J., & Komives, E. A. Site-specific identification and quantitation of endogenous SUMO modifications under native conditions. *Nat. Commun.* **8**, 1171 (2017).

22. Xiao, Z., Chang, J. G., Hendriks, I. A., Sigurdsson, J. O., Olsen, J. V., & Vertegaal, A. C. System-wide analysis of SUMOylation dynamics in response to replication stress reveals novel SUMO target proteins and acceptor lysines relevant for genome stability. *Mol. Cell Proteomics.* **14**, 1419-1434 (2015).

23. Tammsalu, T., Matic, I., Jaffray, E. G., Ibrahim, A. F., Tatham, M. H., & Hay, R. T. Proteome-Wide Identification of SUMO2 Modification Sites. *Sci. Signal.* **7**, rs2 (2014).

24. Lamoliatte, F., Caron, D., Durette, C., Mahrouche, L., Maroui, M. A., Caron-Lizotte, O., Bonneil, E., Chelbi-Alix, M. K., & Thibault, P. Large-scale analysis of lysine SUMOylation by SUMO remnant immunoaffinity profiling. *Nat. Commun.* **5**, 5409 (2014).

25. Minguez, P., Parca, L., Diella, F., Mende, D. R., Kumar, R., Helmer-Citterich, M., Gavin, A. C., van, N., V, & Bork, P. Deciphering a global network of functionally associated post-translational modifications. *Mol. Syst. Biol.* **8**, 599 (2012).

26. Beltrao, P., Bork, P., Krogan, N. J., & van, N., V Evolution and functional cross-talk of protein post-translational modifications. *Mol. Syst. Biol.* **9**, 714 (2013).

27. Vertegaal, A. C. SUMO chains: polymeric signals. *Biochem. Soc. Trans.* **38**, 46-49 (2010).

28. Adorisio, S., Fierabracci, A., Muscari, I., Liberati, A. M., Ayroldi, E., Migliorati, G., Thuy, T. T., Riccardi, C., & Delfino, D. V. SUMO proteins: Guardians of immune system. *J. Autoimmun.*(2017).
