# Supplementary material for: Site-specific characterization of endogenous SUMOylation across species and organs
Source: Nat Commun. 2018 Jun 25;9:2456. doi: 10.1038/s41467-018-04957-4 (PMC6018634; doi:10.1038/s41467-018-04957-4)
Supplement: Supplementary file 3 — Description of Additional Supplementary files [file 41467_2018_4957_MOESM3_ESM.pdf]

Description of Additional Supplementary Files

File Name: Supplementary Data 1
Description: A list of all 14,869 endogenous human SUMO2/3 sites identified in this study, complete with qualitative and quantitative information. Two separate tabs contain information about the two pilot studies performed prior to the main experiments, and two further tabs contain statistical information related to Motif-X analysis of SUMO consensus motifs.

File Name: Supplementary Data 2
Description: A list of all 3,870 endogenous human SUMO2/3 target proteins identified in this study, complete with qualitative and quantitative information.

File Name: Supplementary Data 3
Description: A thorough comparison of the endogenous human SUMO2/3 sites identified in this study to 12 other site-specific SUMO proteomics studies, which represent predominantly exogenous screens.

File Name: Supplementary Data 4
Description: A thorough comparison of the endogenous human SUMO2/3 target proteins identified in this study to 13 other SUMO proteomics studies, which represent a mixture of endogenous and exogenous screens performed at the protein-level or site-level. The entire human proteome was used as a background.

File Name: Supplementary Data 5
Description: A list of all 1,963 endogenous and in vivo mouse SUMO2/3 sites identified in this study, complete with qualitative and quantitative information. A separate tabs contains statistical information related to Motif-X analysis of SUMO consensus motifs.

File Name: Supplementary Data 6
Description: A list of all 955 endogenous and in vivo mouse SUMO2/3 target proteins identified in this study, complete with qualitative and quantitative information.

File Name: Supplementary Data 7
Description: Term enrichment analysis comparing globally identified mouse SUMO2/3 target proteins with the tissue background proteome, including statistical information.

File Name: Supplementary Data 8
Description: Organ-specific term enrichment analyses, comparing mouse SUMO2/3 target proteins directly MS/MS-identified by SUMO2/3 sites in specific organ types with the corresponding organ background proteomes. All statistically significant enriched terms were compared across the eight organs, and terms uniquely identified in a specific organ were highlighted.

File Name: Supplementary Data 9
Description: Direct comparison of human and mouse endogenous SUMO2/3 target proteins identified in this study, complete with qualitative SUMO2/3 site information.

File Name: Supplementary Data 10
Description: Term enrichment analyses comparing subsets of identified human SUMO2/3 and mouse SUMO2/3 target proteins, including statistical information. More information regarding the comparisons is included in the header rows.

File Name: Supplementary Data 11
A list of all 526 identified endogenous human SUMO2/3-phospho peptides, complete with qualitative and quantitative information.

File Name: Supplementary Data 12
Description: A list of all 49 identified endogenous and in vivo mouse SUMO2/3- phospho peptides, complete with qualitative and quantitative information.

File Name: Supplementary Data 13
Description: Four fully annotated MS/MS spectra corresponding to endogenous SUMO2/3-modified peptides directly identified by the mass remnant peptide, DVFQQQTGG, which results from full Asp-N cleavage of SUMO2/3. The mass remnant peptide has a monoisotopic mass of 960.4301, and generally remains covalently attached to the lysine in the target peptide during fragmentation with higher-energy collisional disassociation. This cofragmentation of the SUMO2/3 mass remnant yields a unique suite of diagnostic ions for spectral verification. Coloring: y-ions from target peptide (red), b-ions from target peptide (blue), diagnostic b-ions from SUMO2/3 mass remnant (green), doubly-fragmented internal peptides (purple), y-ions, b-ions, and internal fragments with additional atomic losses (yellow), M+H (cyan), unassigned (black). Asterisks indicate neutral loss of the DVFQQQT (b7-SUMO) part of the mass remnant.
