# Supplementary material for: Site-specific characterization of endogenous SUMOylation across species and organs
Source: Nat Commun. 2018 Jun 25;9:2456. doi: 10.1038/s41467-018-04957-4 (PMC6018634; doi:10.1038/s41467-018-04957-4)
Supplement: Supplementary file 16 — Supplementary Data 13 [file 41467_2018_4957_MOESM16_ESM.pdf]

Raw file	Scan	Method	Score	m/z	Gene	SUMO site
QE4_LC12_IAH_SUMO_R3_HEK_AspN_E1_F5	12424	FTMS; HCD	459.9	958.22	TCF12	K-519

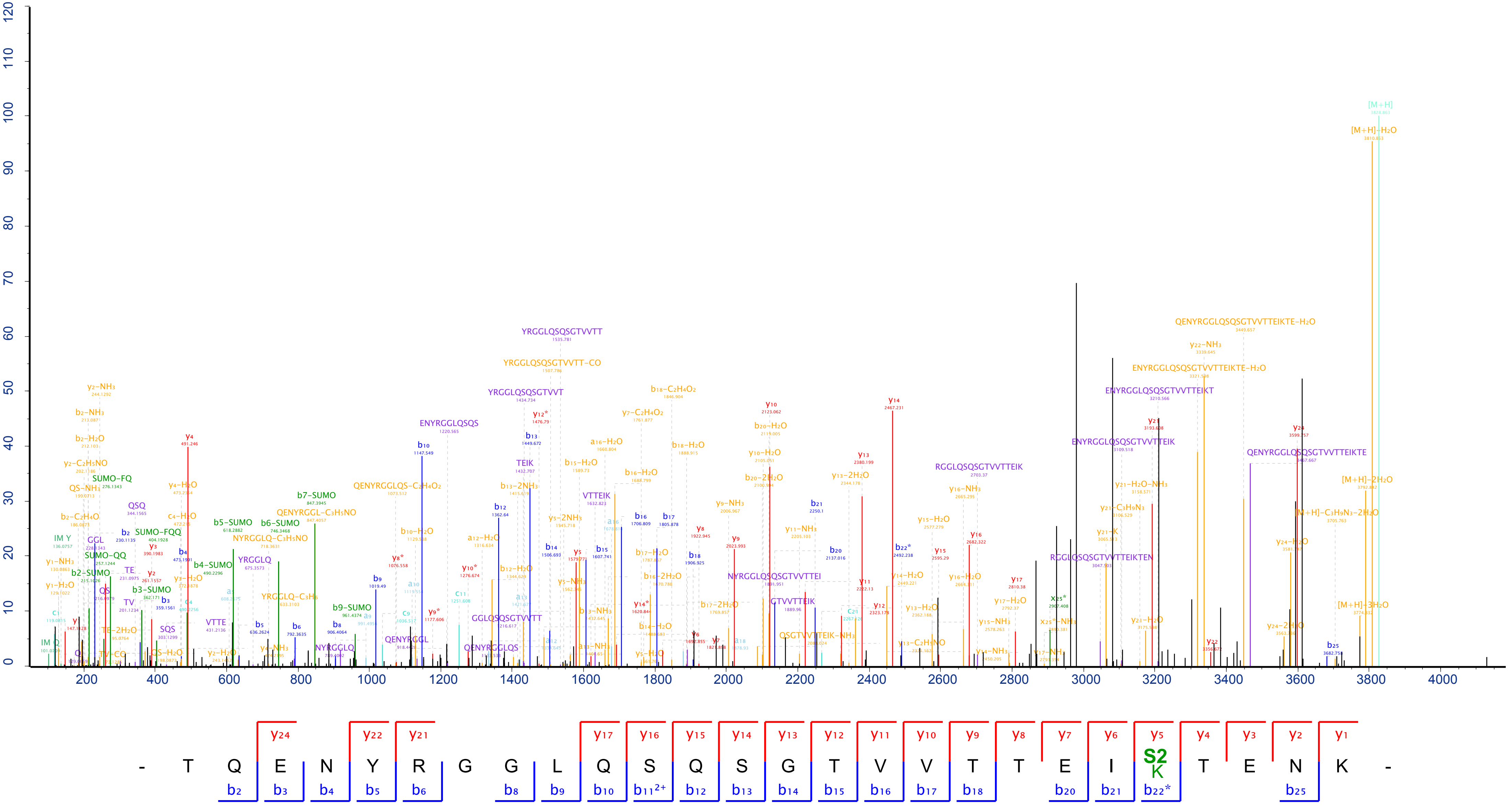

Raw file	Scan	Method	Score	m/z	Gene	SUMO site
QE4_LC12_IAH_SUMO_R3_HEK-H_AspN_E1_F5	16792	FTMS; HCD	486.77	1098.19	TOP2B	K-28

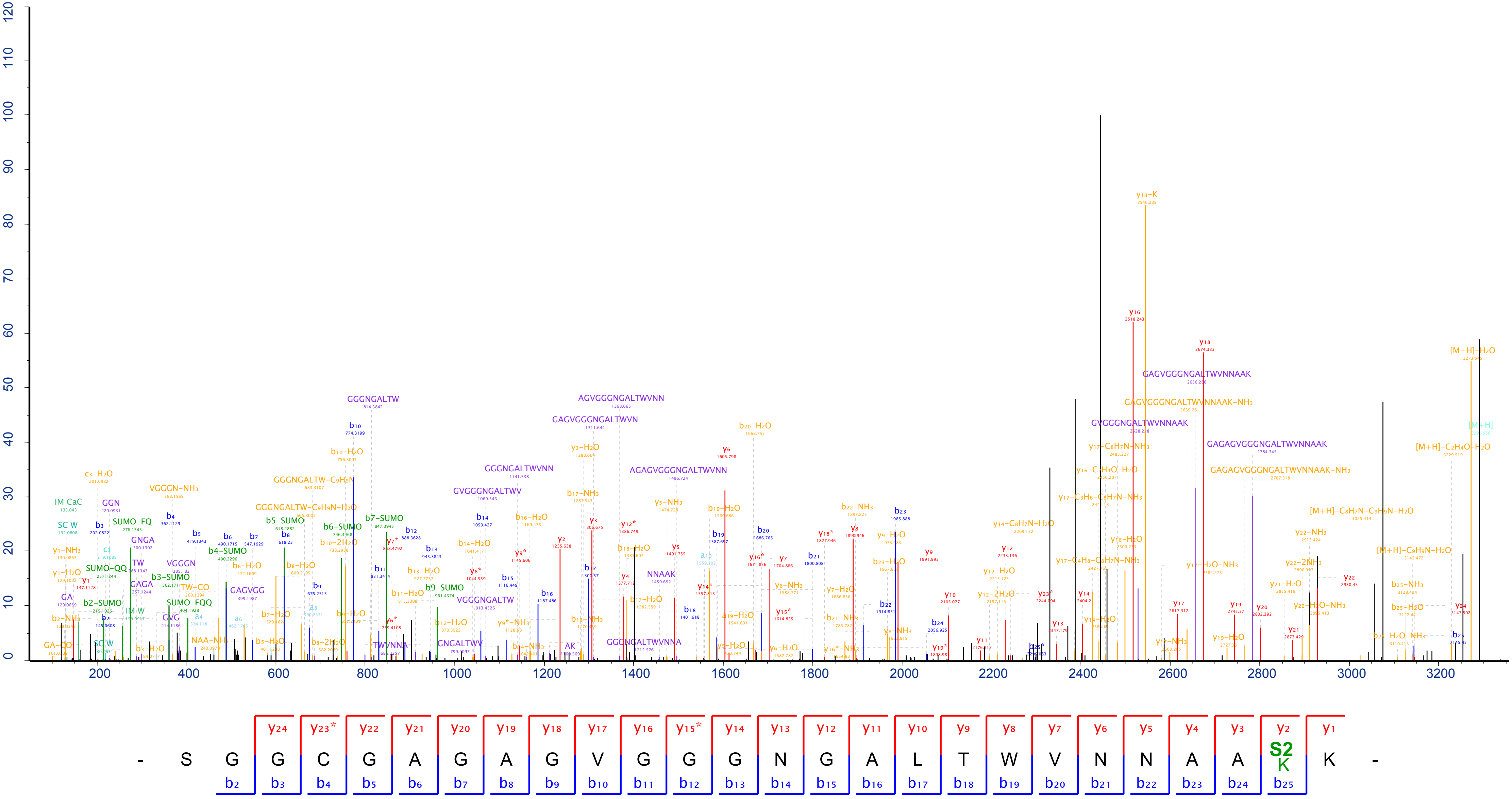

Supplementary Data 13 (2/4)

Raw file	Scan	Method	Score	m/z	Gene	SUMO site
QE4_LC12_IAH_SUMO_R3_HEK_AspN_E2_F5	25150	FTMS; HCD	477.36	1292.28	ZMYM4	K-250

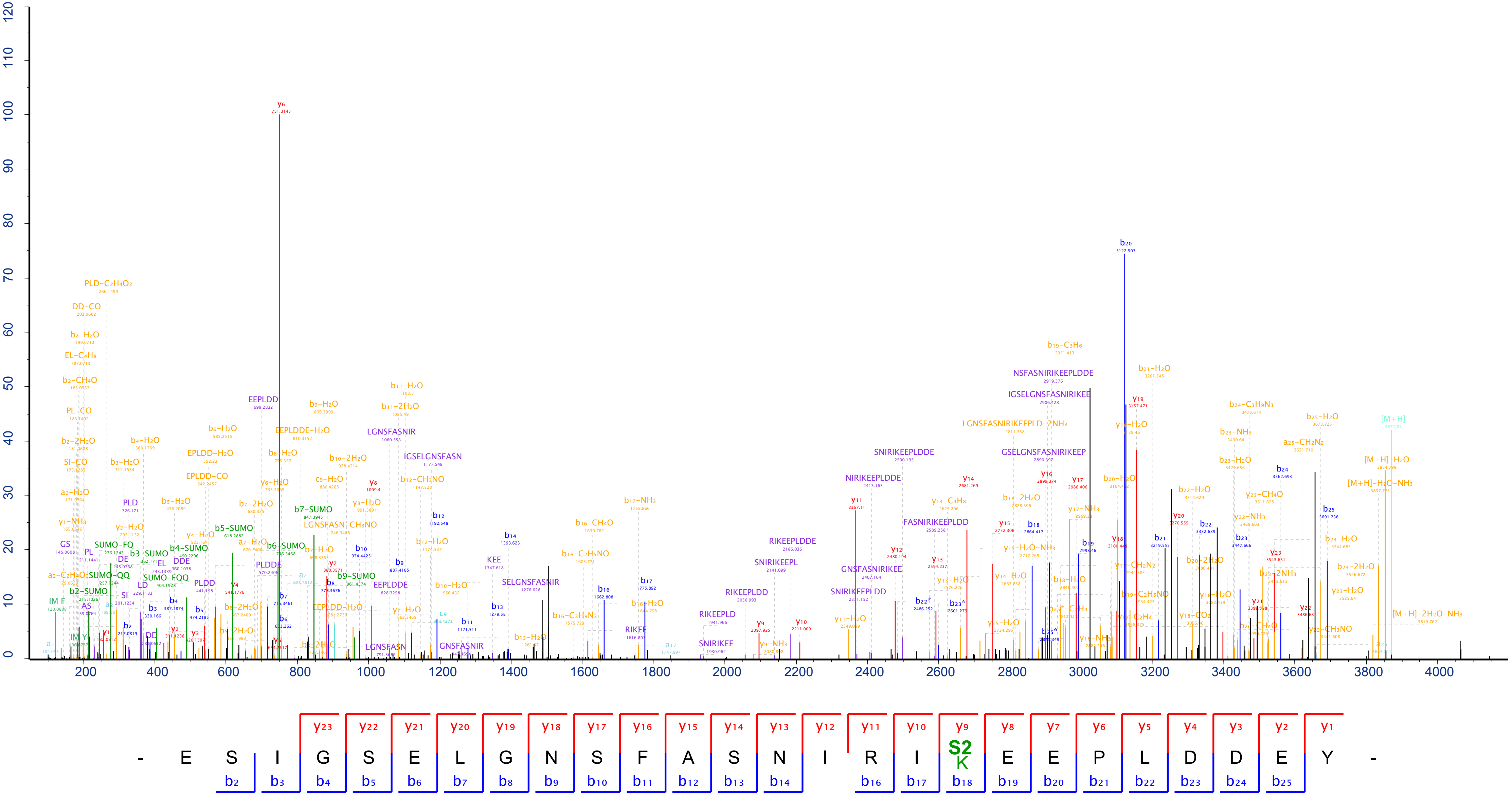

Supplementary Data 13 (3/4)

Raw file	Scan	Method	Score	m/z	Gene	SUMO site
QE4_LC12_IAH_SUMO_R3_HEK-H_AspN_E1_F1	27572	FTMS; HCD	464.1	983.97	HNRNPM	K-322

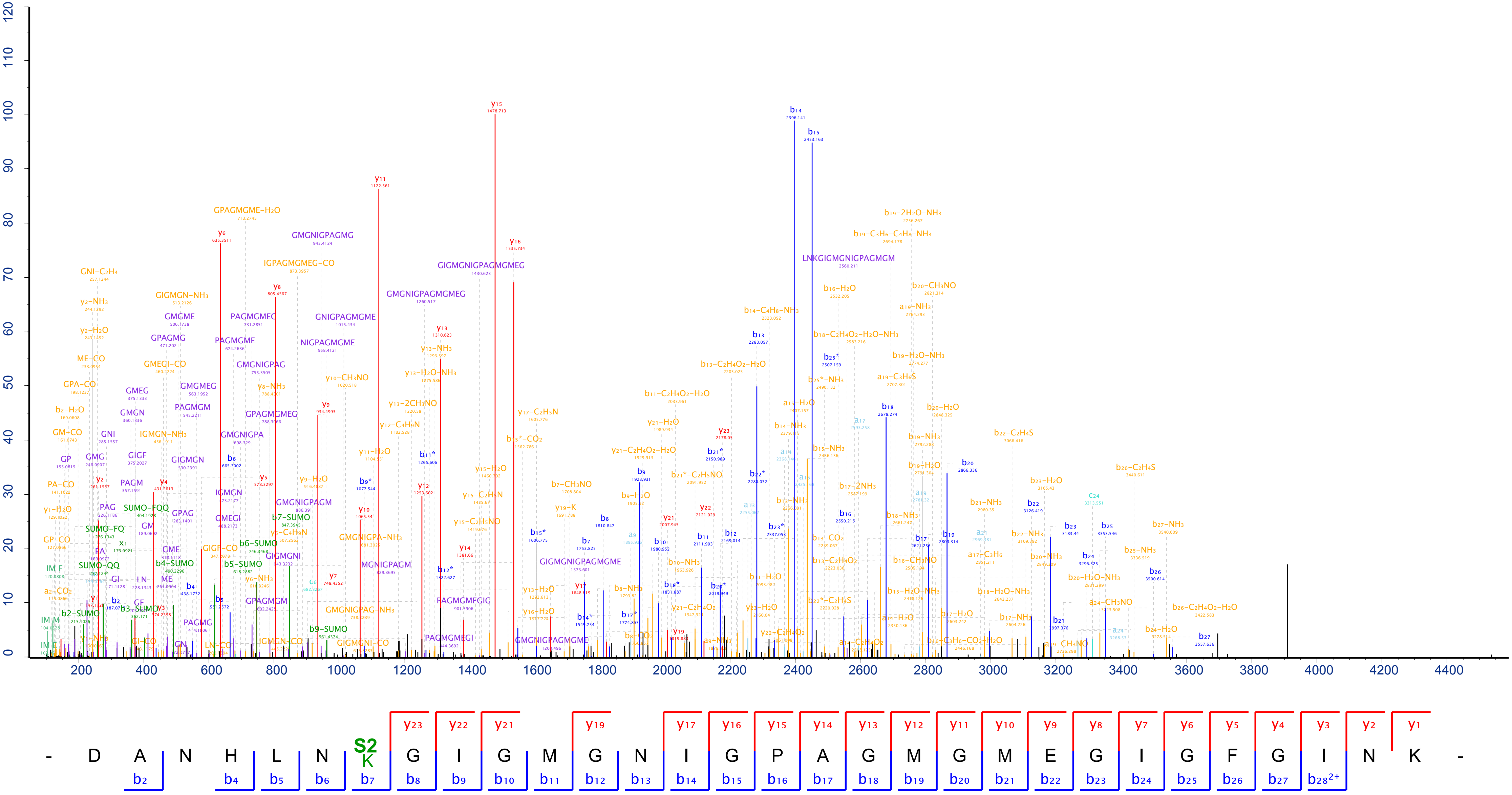

Supplementary Data 13 (4/4)
